# Atomic Pt‐Promoted Hierarchical CoP@CNTs‐Bridged CeO_2_–CoP Heterostructure‐Based Hollow Microcubes for Water Electrolysis

**DOI:** 10.1002/smll.202507407

**Published:** 2025-10-14

**Authors:** Xue Li, Kaixuan Dong, Saleem Sidra, Do Hwan Kim, Duy Thanh Tran, Nam Hoon Kim, Joong Hee Lee

**Affiliations:** ^1^ Department of Nano Convergence Engineering Jeonbuk National University Jeonju Jeonbuk 54896 Republic of Korea; ^2^ Division of Science Education Graduate School of Department of Energy Storage/Conversion Engineering Jeonbuk National University Jeonju Jeonbuk 54896 Republic of Korea; ^3^ AHES Co. 445 Techno Valley‐ro, Bongdong‐eup Wanju‐gun Jeonbuk Republic of Korea; ^4^ Carbon Composite Research Center Department of Polymer−Nano Science and Technology Jeonbuk National University Jeonju Jeonbuk 54896 Republic of Korea

**Keywords:** CeO_2_–CoP heterostructure, hierarchical CoP@CNTs bridging, overall water splitting, Pt single atoms

## Abstract

In this work, the electrocatalyst systems of CoP nanoparticles confined within carbon nanotubes are presented, which bridge CeO_2_–CoP heterostructures embedded in nitrogen‐doped hollow carbon nanocubes (CoP@CNTs/CeO_2_–CoP@NCs) and further engineered with Pt single atom (Pt–CoP@CNTs/CeO_2_–CoP@NCs). The Pt–CoP@CNTs/CeO_2_–CoP@NCs catalyst exhibits outstanding performance for hydrogen and oxygen evolution in 1.0 m KOH medium. The two‐electrode Pt–CoP@CNTs/CeO_2_–CoP@NCs_(−)_||CoP@CNTs/CeO_2_–CoP@NCs_(+)_ configuration reaches a low cell voltage of 1.52 V in 30 wt.% KOH and 1.53 V in 1.0 m KOH. This system further demonstrates exceptionally high mass activity, ≈38.5‐fold greater than that of commercial Pt/C_(−)_||RuO_2(+)_. The fabricated anion exchange membrane electrolyzer stack provides a stack voltage of 1.71/1.84/2.04 V at 0.5/1/2 A cm^−2^ in 1.0 m KOH at 60 °C and excellent stability for over 1400 h. The CeO_2_–CoP heterostructures‐sealed nitrogen‐doped hollow carbon nanocubes‐bridged CoP@CNTs architecture owns a hierarchical structure with numerous metallic heterogeneous interfaces and efficient connection between different active phases to ensure abundant active sites, superb conductivity, short electron transfer pathways, and controlled adsorption/desorption of intermediates for rapid OER kinetics. In addition, the incorporation of a small Pt amount into CoP@CNTs/CeO_2_–CoP@NCs generates unique electronic properties to reach high catalytic HER performance, thereby yielding the efficient electrocatalyst systems for sustainable and economically viable water splitting.

## Graphical abstract

The electrocatalyst systems of CoP nanoparticles confined within CNTs, which bridge CeO_2_–CoP heterostructures embedded in N‐doped hollow carbon nanocubes and further engineered with Pt single atom exhibit outstanding performance for HER and OER in 1.0 m KOH. The Pt–CoP@CNTs/CeO_2_–CoP@NCs_(−)_||CoP@CNTs/CeO_2_–CoP@NCs_(+)_‐based AEMWE stack provides stack voltage of 1.71/1.84/2.04 V at 0.5/1/2 A cm^−2^ in 1.0 m KOH at 60 °C and excellent stability for over 1400 h.

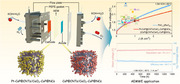

## 1 Introduction

Hydrogen is widely regarded as a promising alternative to fossil fuels due to its high energy density (142.4 MJ kg^−1^), low weight, and environmentally friendly energy characteristics, as it produces no greenhouse gas emissions during use.^[^ ^1^, ^2^ ^]^ Among the established production methods, green hydrogen generated through electrocatalytic water splitting stands out for its sustainability since it produces zero emissions during both its generation and application.^[^ ^3^ ^]^ Despite these advantages, water splitting remains costly,^[^ ^4^, ^5^ ^]^ primarily due to the slow kinetics associated with the hydrogen evolution reaction (HER) and oxygen evolution reaction (OER), which pose considerable barriers to widespread implementation.^[^ ^6^, ^7^ ^]^ Although noble metals such as Pt/C and RuO_2_ serve as highly active catalysts for HER and OER, their use substantially elevates the overall production cost.^[^ ^8^, ^9^ ^]^ As the demand for green hydrogen increases, the development of high‐performance, sustainable, and low‐cost water‐splitting systems is becoming more critical. Accordingly, researchers have focused on developing new electrocatalyst materials to reduce the overpotential associated with overall water‐splitting (OWS).^[^ ^10^, ^11^, ^12^, ^13^, ^14^, ^15^ ^]^ Cobalt‐based catalysts are particularly notable for OER because their available empty orbitals and abundant d‐electrons promote crucial adsorption and desorption reactions. Cobalt phosphides, particularly, exhibit high conductivity and generate diverse active intermediates during the OER process, including Co_x_P/Co─O, Co_x_P/Co─OH, and Co_x_P/Co─OOH, all contributing to enhanced electrocatalytic activity.^[^ ^16^, ^17^ ^]^ Nonetheless, the overpotential remains excessively high for viable large‐scale implementation, emphasizing the urgent need for catalyst optimization. Cerium oxide (CeO_2_), another promising candidate, benefits from its stable redox cycles (Ce^3+^/Ce^4+^), presence of oxygen vacancies, robust oxygen binding, and distinctive chemical characteristics. Guo et al. fabricated a CeO_2_@CoSe_2_/CC electrode with improved surface reconstruction and engineered interfaces, realizing efficient water splitting at low overpotentials (245 mV for OER, 138 mV for HER) and an electrolysis voltage of 1.54 V, thereby providing guidance for next‐generation catalyst development.^[^ ^18^ ^]^ Constructing a heterostructure of CeO_2_ with CoP further enhances OER catalytic activity by increasing the density of active sites and facilitating charge transport. Pt‐based catalysts still represent the benchmark for HER performance owing to their superior activity, but the high cost restricts their broad adoption. To address this challenge, single‐atom catalysts (SACs) have been introduced to minimize noble metal content while preserving high atomic efficiency and activity.^[^ ^19^ ^]^ Strategic selection of support materials is critical for stabilizing Pt single atoms and inhibiting their aggregation. Both CoP and CeO_2_ have shown strong interactions with isolated Pt atoms, resulting in robust and durable catalytic centers.^[^ ^20^ ^]^ Ye et al. developed a single‐atom Pt catalyst embedded in mesoporous CoP nanosheets on carbon fiber cloth (Pt_at_–CoP MNSs/CFC) with ultralow Pt loading (0.7 wt.%) was developed, exhibiting outstanding HER performance in both alkaline and seawater electrolysis. This catalyst demonstrates a negligible onset potential, elevated catalytic activity, and superior durability when compared to commercial Pt/C catalysts, attributed to robust Pt–CoP interactions that promote spontaneous water dissociation.^[^ ^21^ ^]^ Furthermore, MOF derivatives such as carbon nanotubes derived from MOFs are emerging as promising catalysts for electrocatalysis, owing to their robust and conductive networks, which have attracted significant research interest. Yan et al. prepared a freestanding 3D heterostructure film consisting of a Ni‐centered MOF and graphene oxide; subsequent pyrolysis yields 1D carbon nanotubes (CNTs) integrated with 2D reduced graphene oxide sheets. The combination of N‐doped carbon shells and Ni nanoparticles contributes to excellent electrocatalytic activity, achieving low overpotentials for HER (95 mV) and OER (260 mV) at 10 mA cm^−2^.^[^ ^22^ ^]^ Employing highly efficient support materials such as CNTs results in a 3D hybrid architecture with improved connectivity due to the linking properties of flexible and highly conductive CNTs, forming a stable network.^[^ ^23^ ^]^ This structure promotes rapid charge transfer and establishes multiple channels for ion and electron transport, thereby markedly enhancing catalytic activity.

Building on the described catalyst engineering principles, we developed a novel catalyst engineering approach involving the self‐templated growth of a Co/Ce bimetallic ZIF, followed by selective phosphidization and Pt single‐atom implantation, to synthesize high‐efficiency CoP@CNTs/CeO_2_–CoP@NCs for OER and Pt–CoP@CNTs/CeO_2_–CoP@NCs for HER. These advanced catalysts display markedly reduced overpotentials for HER and OER, as well as outstanding performance in fabricated electrolyzers. The electrolyzer assembled from Pt–CoP@CNTs/CeO_2_–CoP@NCs_(−)_||CoP@CNTs/CeO_2_–CoP@NCs_(+)_ demonstrated low cell voltages in alkaline media while maintaining stable operation, with minimal performance decay. Notably, the AEMWE stack integrating Pt–CoP@CNTs/CeO_2_–CoP@NCs_(−)_||CoP@CNTs/CeO_2_–CoP@NCs_(+)_ achieved stack voltages of only 1.71, 1.84, and 2.04 V for current densities of 0.5, 1, and 2 A cm^−2^, respectively, while sustaining excellent durability over 1400 h of continuous operation. Altogether, these results highlight the promise of the synthesized materials as effective catalysts for practical water‐splitting applications and their contribution to advancing green hydrogen production technologies.

## 2 Results and Discussion

**Scheme 1:**
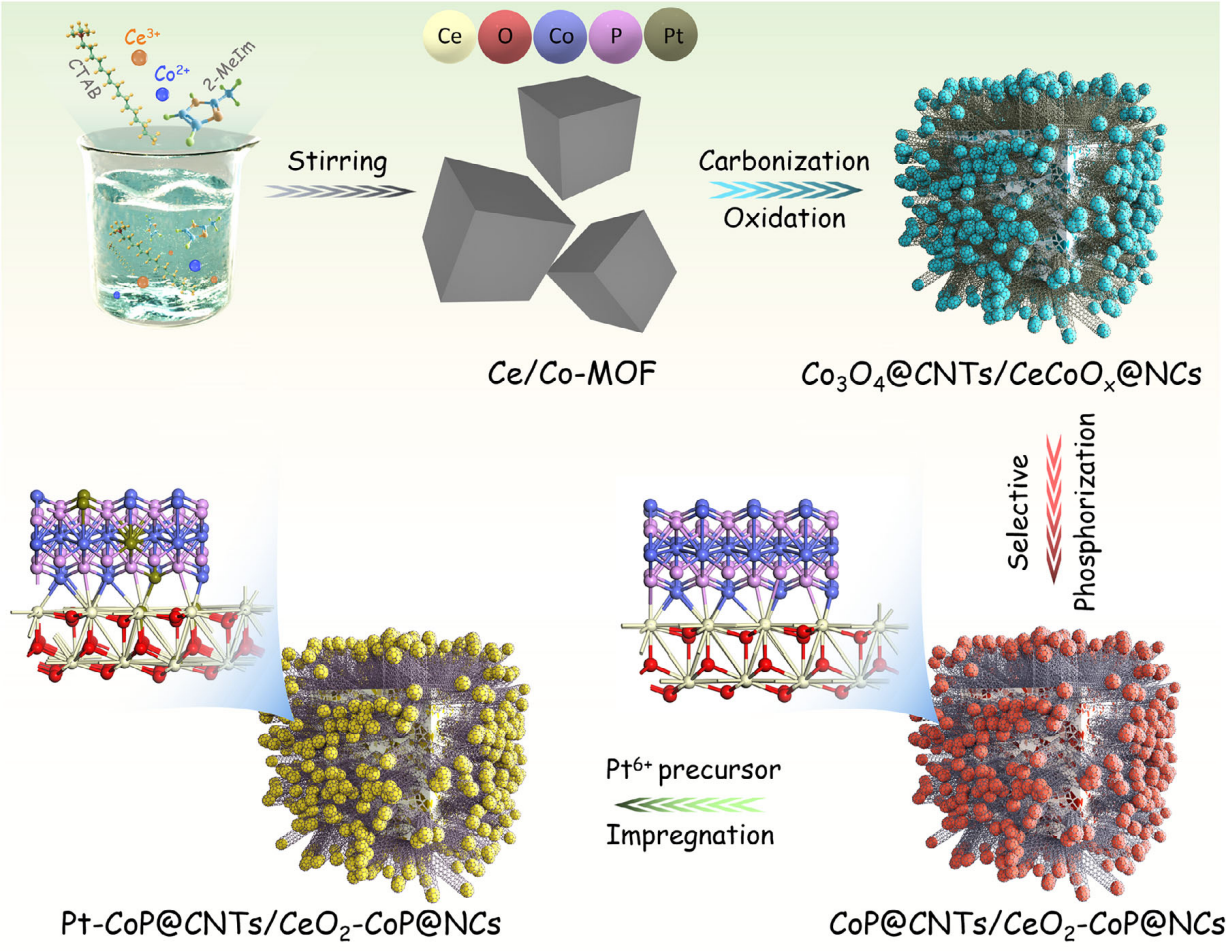
Schematic illustration of the fabrication for Pt–CoP@CNTs/CeO_2_–CoP@NCs hybrid material.

The synthesis process, as outlined in **Scheme** **1**, entails the combination of Ce(NO_3_)_3_ and Co(NO_3_)_2_ metal precursors with the organic ligand 2‐methylimidazole and the surfactant cetyl trimethyl ammonium bromide (CTAB), followed by stirring in deionized water. This method results in the formation of a Ce‐incorporated zeolitic imidazolate framework‐67 (CeCo–MOF) that exhibits a well‐defined cubic morphology and an average particle size near 300 nm (Figure S1a,b, Supporting Information). Energy‐dispersive X‐ray spectroscopy (EDS) mapping demonstrates a uniform distribution of Co, Ce, O, C, and N within CeCo−MOF, thereby confirming the successful incorporation of Ce into the Co–MOF (ZIF‐67) matrix (Figure S1c–e, Supporting Information). The X‐ray diffraction (XRD) analysis of CeCo–MOF shows diffraction patterns that match those of the standard Co–MOF (ZIF‐67), validating the retention of the original crystal structure following Ce incorporation (Figure S2, Supporting Information). Subsequent thermal treatment in an Ar/H_2_ (90/10) environment at 800 °C, followed by exposure to air, induces dual transformations: Co nanoparticles (Co NPs) catalyze the extensive growth of carbon nanotubes (CNTs) on the particle surfaces, and oxidized Ce/Co NPs yield CeCoO_x_ (Figure S3a,b, Supporting Information). The formation of a dense CNT network on the surface significantly increases electrical conductivity by providing effective conductive channels.^[^ ^23^ ^]^ Additional EDS mapping confirms that Co, Ce, O, C, and N elements are uniformly distributed throughout the Co_3_O_4_@CNTs/CeCoO_x_@NCs heterostructure (Figure S3c–e, Supporting Information). The subsequent phosphidization process at low temperature selectively transforms Co_3_O_4_ into CoP, while preserving the overall structural framework (Figure S4a,b, Supporting Information), resulting in the production of CoP@CNTs/CeO_2_–CoP@NCs. EDS color mapping indicates homogeneous dispersion of Co, Ce, P, O, C, and N elements across the entire CoP@CNTs/CeO_2_–CoP@NCs heterostructure (Figure S4c–f, Supporting Information). To further optimize the electronic structure of the CeO_2_–CoP heterostructure and harness its abundant electroactive sites, Pt single atoms (SAs) were introduced into the CeO_2_–CoP framework via immersion in a Pt^6+^ precursor solution, yielding Pt–CoP@CNTs/CeO_2_–CoP@NCs. CeO_2_ is notable for its ability to accommodate oxygen vacancies and structural defects, which serve as electron donors, establishing a localized reductive environment.

**Figure 1:**
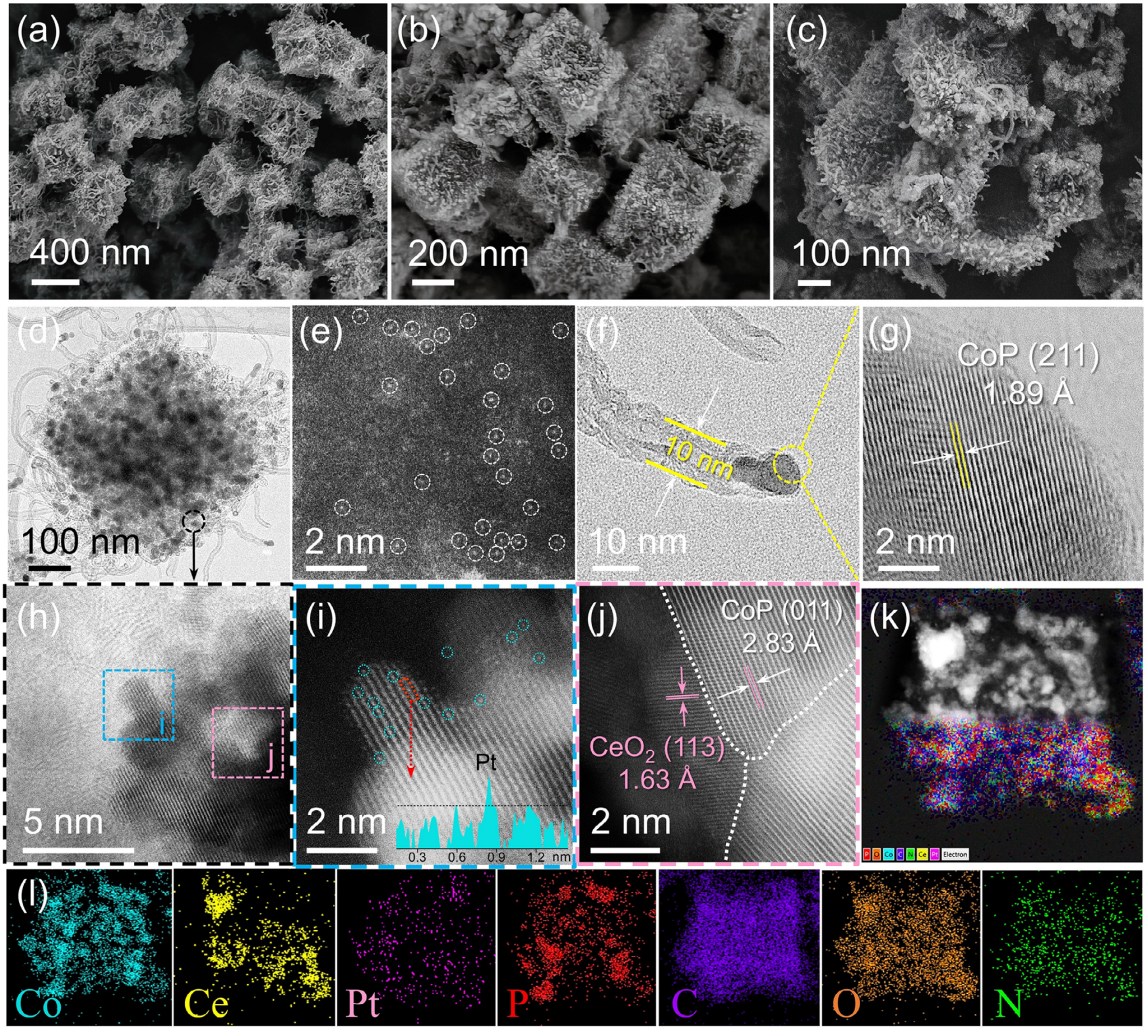
a–c) FE‐SEM images, d) TEM image of Pt–CoP@CNTs/CeO_2_–CoP@NCs; e) HR‐TEM image of Pt–CoP@CNTs/CeO_2_–CoP@NCs; f,g) HR‐TEM images of CNT structure; h) HR‐TEM image of Pt–CoP@CNTs/CeO_2_–CoP@NCs along with the corresponding area of i) HAADF‐STEM image at a thin area (Inset: Line profile intensity), j) HAADF‐STEM image showing crystalline features of heterostructure at a distinct interface; k,l) STEM and EDS color mapping images of Pt–CoP@CNTs/CeO_2_–CoP@NCs.

When the Pt^6+^ precursor is introduced to hybrid structure, the existing anion and cation vacancy defects on surface of CeO_2_ and CoP display as reductive sites to reduce Pt^6+^ to lower oxidation states, which facilitates the immobilization of Pt atoms at the defect sites.^[^ ^24^, ^25^ ^]^ Especially, the formation of CeO_2_–CoP heterostructure results in a multi‐interfacial state possessing the enrichment of cation and anion vacancy defects, which are very unstable and reactive to promote the reduction of Pt^4+^ into Pt SAs without the necessity of an additional reducing agent.^[^ ^26^, ^27^ ^]^ As depicted in **Figure** **1**a,b, the Pt–CoP@CNTs/CeO_2_–CoP@NCs sample preserves its microstructure without obvious deterioration, similar to that of CoP@CNTs/CeO_2_–CoP@NCs and Co_3_O_4_@CNTs/CeCoO_x_@NCs. During thermal treatment, outward cobalt migration leads to ZIF framework decomposition, generating voids, while concurrent oxidation of cerium forms CeO_2_, thus maintaining the hollow nanostructure as explained by the Kirkendall effect.^[^ ^27^ ^]^ After Pt implantation, the hollow nanostructure is largely preserved, as confirmed by the SEM image in Figures 1c. 1d provides a representative TEM image of Pt–CoP@CNTs/CeO_2_–CoP@NCs, displaying a unique configuration where CNT‐bridged nanocubes incorporate multiple NPs. Notably, the HR‐TEM image in Figure 1e displays nanocubes densely populated by Pt SA sites, distinguishable as bright dots encircled by a white dashed line. Figure 1f,g depict CNTs with a small diameter ≈10 nm, exhibiting clear multiwall structures and CoP NPs encapsulated within a carbon layer at the tip. These hierarchical CNT layers act as conductive bridges, which markedly enhance electrical conductivity.^[^ ^28^ ^]^ The HR‐TEM image in Figure 1h offers detailed information on an enlarged region from Figure 1d. A thin section, indicated by a blue dashed box at the edge of the nanocube, displays distinct lattice fringes, where the presence of Pt SAs is more frequently detected. The high‐angle annular dark‐field scanning TEM (HAADF‐STEM) image, performed at the Center for University‐wide Research Facilities (CURF), Jeonbuk National University (JBNU), in Figure 1i shows multiple bright dots, encircled in blue, which are attributed to Pt SA sites because of their higher atomic weight relative to Co or Ce. The line scan profile of the atomic columns in the red dashed box of provides further evidence for the existence of Pt SAs, as the increased intensity indicates the incorporation of Pt atoms with a larger atomic radius.^[^ ^29^ ^]^ Figure 1j demonstrates the CeO_2_–CoP heterostructure, illustrating a distinct interfacial area with observed lattice fringes of 0.283 and 0.163 nm, which are assigned to the (011) facet of CoP and the (113) facet of CeO_2_, respectively. Figure 1k,l displays the STEM and EDS color mapping of Pt–CoP@CNTs/CeO_2_–CoP@NCs, demonstrating the homogenous distribution of Co, Ce, P, O, C, N, and Pt elements throughout the nanostructure. Their respective concentrations, ≈17.15, 8.34, 3.88, 10.1, 57.64, 2.8, and 1.41 wt.%, further confirm the successful synthesis of Pt–CoP@CNTs/CeO_2_–CoP@NCs. By adjusting the concentration of Pt precursors during synthesis, Pt–CoP@CNTs/CeO_2_–CoP@NCs materials containing varying Pt contents were prepared (Figures S5 and S6, Supporting Information). Catalytic activity tests establish that utilizing 0.3 mm H_2_PtCl_6_ yields the best results (Figure S7, Supporting Information). For reference, a Pt–Co_3_O_4_@CNTs/CeCoO_x_@NCs sample was also produced. As illustrated in Figure S8 (Supporting Information), the material maintains morphology and structural characteristics comparable to Pt–CoP@CNTs/CeO_2_–CoP@NCs and Co_3_O_4_@CNTs/CeCoO_x_@NCs, confirming its structural durability.

**Figure 2:**
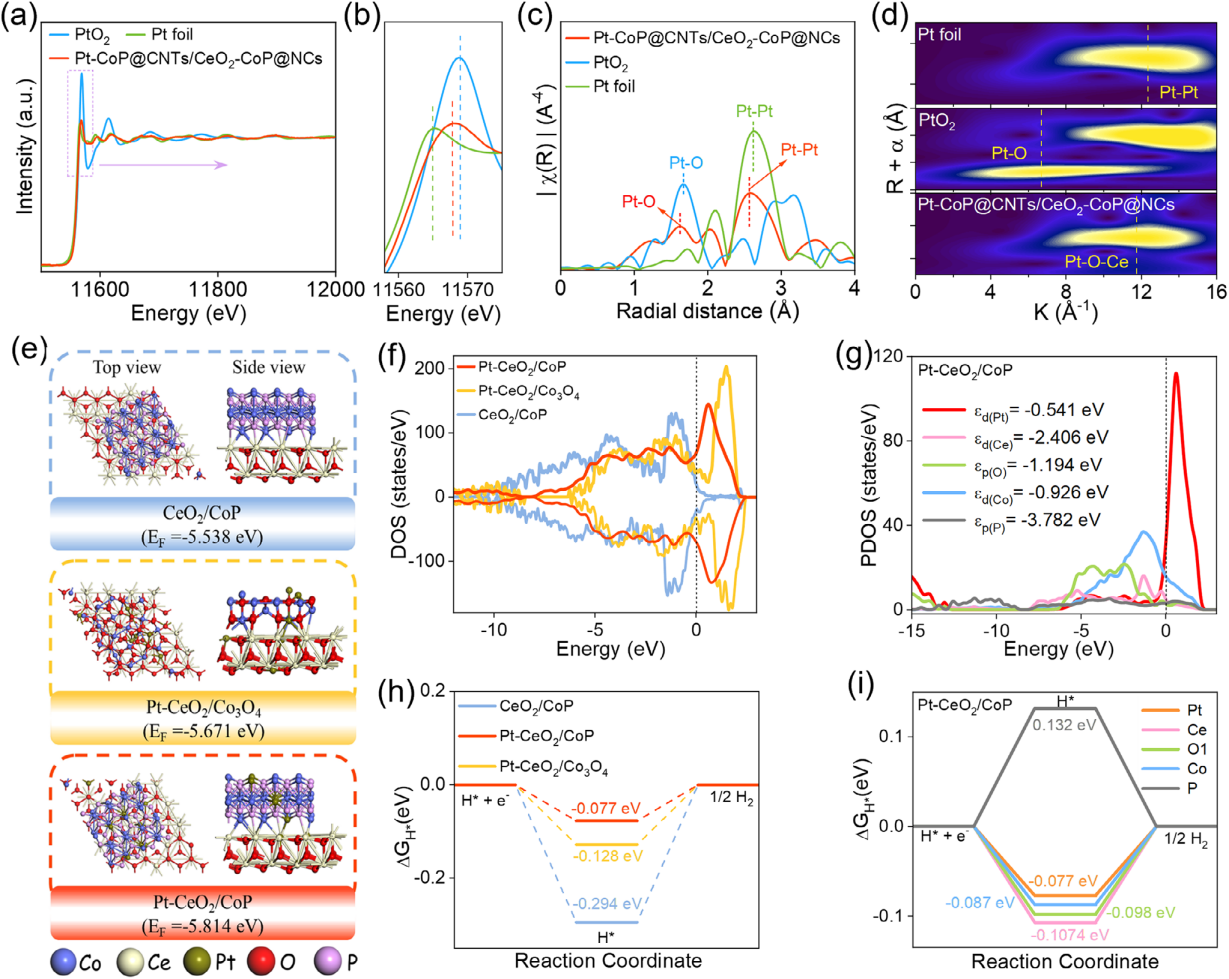
a) XANES and b) high‐magnification XANES spectra, c) FT‐EXAFS spectra, and d) WT EXAFS from Pt–CoP@CNTs/CeO_2_–CoP@NCs, Pt foil, and PtO_2_; e) Structural models of different materials used for DFT calculation; f) DOS of different structural models; g) PDOS of different elements in Pt–CeO_2_/CoP model; h) ΔG_H*_ of different materials; i) ΔG_H*_ at different active sites on surface of the Pt–CeO_2_/CoP model.

To determine the presence of Pt as SAs on the Pt–CoP@CNTs/CeO_2_–CoP@NCs architecture, X‐ray absorption spectroscopy (XAS) was employed to examine both the oxidation state and the local coordination environment of Pt in the composite. As displayed in **Figure** **2**a,b, the X‐ray absorption near‐edge structure (XANES) spectra for Pt–CoP@CNTs/CeO_2_–CoP@NCs, together with those of Pt foil and PtO_2_ as references, show that the absorption edge for Pt–CoP@CNTs/CeO_2_–CoP@NCs is located between that of metallic Pt and PtO_2_. This indicates that Pt in the composite exhibits a mixed valence state within the range of 0 and + 4. The results from the extended X‐ray absorption fine structure (EXAFS) analysis, provided in Figure 2c, confirm the presence of coordination signals consistent with Pt─O and Pt─O─Ce bonds, as previously documented. Additionally, the wavelet transform (WT) analysis of EXAFS data (Figure 2d) underscores a characteristic spatial distribution of Pt─O─Ce interactions in Pt–CoP@CNTs/CeO_2_–CoP@NCs, which differ from those observed in PtO_2_ and Pt foil, indicating a uniquely unsaturated coordination environment and robust metal–support interactions.^[^ ^30^ ^]^ The orthorhombic unit cell of CoP (JCPDS #03‐065‐2593) in the space group ^*^Pnma^*^ was optimized using a 7 × 7 × 7 Monkhorst–Pack k‐point grid for Brillouin zone sampling. Similarly, the cubic unit cells of CeO_2_ (JCPDS #98‐016‐9030) and Co_3_O_4_ (JCPDS #98‐006‐9375) in space groups Fm‐3m and Fd‐3m, respectively, were optimized with the same 7 × 7 × 7 Monkhorst–Pack k‐point grid for Brillouin zone sampling. We then constructed surfaces for CoP, CeO_2_, and Co_3_O_4_ along specific crystallographic planes (211), (111), and (113), respectively with a 20 Å vacuum applied along the z‐direction. The CeO_2_/CoP, Pt–CeO_2_/CoP, and Pt–CeO_2_/Co_3_O_4_ heterostructures were designed based on experimental data (Figure 2e). During structural optimization, half of the layers in structures were allowed to relax, while the remaining half‐layers were kept fixed. The density of states (DOS) and the Gibbs free energy change of hydrogen adsorption (ΔG_H*_) are critical parameters for evaluating electrocatalyst performance. A higher DOS near the Fermi level generally indicates enhanced conductivity, while an ideal HER catalyst exhibits a ΔG^H*^ value close to zero (0 eV). To assess these properties, we calculated both the DOS and ΔG_H*_ of the materials. As shown in Figure 2f, Pt–CeO_2_/CoP exhibits a significantly higher spin‐polarized DOS near the Fermi level compared with CeO_2_/CoP and Pt–CeO_2_/Co_3_O_4_. This suggests that the Pt–CeO_2_/CoP heterostructure possesses more available electronic states near the Fermi level, leading to abundant charge carriers, improved mobility, and superior conductivity, which collectively enhance catalytic activity. In addition, the high intensity and broadened state of upshifted d‐band center (ε_d_) derived from Pt and then Co d‐orbitals implies their significant contribution to improve adsorption strength and enhance catalytic behavior by strengthening interactions between catalyst and reactant intermediates (Figure 2g). Furthermore, Figure 2h shows that the ΔG_H*_ of the Pt–CeO_2_/CoP structure is found to be −0.077 eV, which is closer to 0.0 eV than those of the CeO_2_/CoP (−0.128 eV) and Pt–CeO_2_/Co_3_O_4_ (−0.294 eV), suggesting its superior properties for promoting the HER process. To further identify the main catalytic active sites over the surface of the Pt–CeO_2_/CoP, the adsorption of a hydrogen atom on different atomic positions is investigated in Figure 2i. The calculated ΔG_H*_ values is found to be −0.077 eV for Pt, −0.107 eV for Ce, −0.098 eV for O, −0.087 eV for Co, and 0.132 eV for P. Among these, ΔG_H*_ value at the Pt site is closest to the ideal 0.00 eV, confirming that Pt atoms serve as the most favorable active centers in Pt–CeO_2_/CoP heterostructure to offer outstanding HER performance. In another regard, the OER Gibbs free energy analysis of CeO_2_/CoP and Pt–CeO_2_/CoP in Figures S9–S11 (Supporting Information) demonstrates that upon the incorporation of Pt into CeO_2_/CoP, the overall OER performance decreases, as reflected by the increased overpotential at all active site on surface of the Pt–CeO_2_/CoP as compared to those of CeO_2_/CoP. Although Pt doping increases the DOS near the Fermi level and improves conductivity of the heterostructure, the OER free‐energy profiles reveal that Pt not only exhibits unfavorable behavior to OER but also causes a negative effect on the local electronic environment of Ce, O, Co, and P sites in Pt–CeO_2_/CoP to resulting in a much higher barrier of catalytic properties with large overpotential towards OER process. The intrinsic activity of CeO_2_/CoP towards OER is mainly originates from Co sites, which show the lowest overpotential of 0.47 V, with the ^*^O → ^*^OOH transition identified as the rate‐determining step. Other sites, including Ce (0.54 V), P (0.83 V), and O (1.00 V), exhibit higher barriers and thus possessing weaker catalytic contributions.

**Figure 3:**
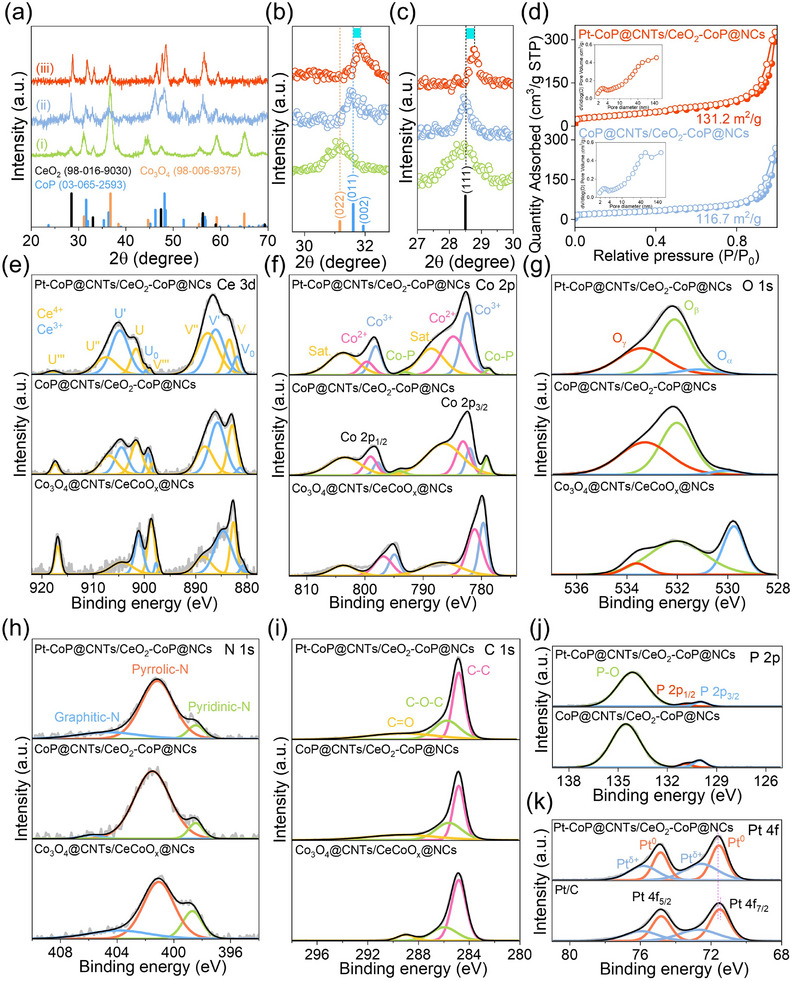
a) XRD patterns of (iii) Pt–CoP@CNTs/CeO_2_–CoP@NCs, (ii) CoP@CNTs/CeO_2_–CoP@NCs, and (i) Co_3_O_4_@CNTs/CeCoO_x_@NCs; b) High‐magnification XRD patterns of Co_3_O_4_ and CoP at 2θ range (29.6–32.8°); c) High‐magnification XRD patterns of CeO_2_ on various materials at the 2θ range (27–30°); d) N_2_ adsorption–desorption isotherms (Inset: pore‐size distribution) of Pt–CoP@CNTs/CeO_2_–CoP@NCs and CoP@CNTs/CeO_2_–CoP@NCs; High‐resolution XPS spectra of e) Ce 3d, f) Co 2p, g) O 1s, h) N 1s, and i) C 1s from Pt–CoP@CNTs/CeO_2_–CoP@NCs, CoP@CNTs/CeO_2_–CoP@NCs, and Co_3_O_4_@CNTs/CeCoO_x_@NCs; High‐resolution XPS spectra of j) P 2p binding energy from Pt–CoP@CNTs/CeO_2_–CoP@NCs and CoP@CNTs/CeO_2_–CoP@NCs, and k) Pt 4f binding energy from Pt–CoP@CNTs/CeO_2_–CoP@NCs and Pt/C.

The crystalline properties of Pt‐CoP@CNTs/CeO_2_‐CoP@NCs, CoP@CNTs/CeO_2_–CoP@NCs, and Co_3_O_4_@CNTs/CeCoO_x_@NCs catalysts were evaluated by XRD analysis, as presented in **Figure** **3**a. The diffraction peaks associated with CeO_2_ (PDF# 98‐016‐9030), CoP (PDF# 03‐065‐2593), and Co_3_O_4_ (PDF# 98‐006‐9375) are distinctly identified. Specifically, peaks at 2θ values of 28.5, 33.0, 47.4, 56.2, and 60.0° correspond to the (111), (002), (022), (113), and (222) crystal planes of CeO_2_. For CoP, diffraction peaks at 31.6, 36.3, 46.2, 48.1, 52.2, and 61.6° are indexed to the (011), (111), (112), (211), (103), and (203) planes, respectively. The Co_3_O_4_ exhibits characteristic peaks at 31.2, 36.7, 38.4, 44.7, 59.1, and 65.6°, which correspond to the (022), (113), (222), (004), (115), and (044) planes. The XRD pattern for Co_3_O_4_@CNTs/CeCoO_x_@NCs displays a prominent peak at 31.2° (Co_3_O_4_ (022)), which vanishes in CoP@CNTs/CeO_2_–CoP@NCs after phosphidization, thus confirming the full conversion of Co_3_O_4_ to CoP (Figure 3b). For Pt–CoP@CNTs/CeO_2_–CoP@NCs, no peaks related to Pt clusters or nanocrystals are detected, demonstrating that Pt is atomically dispersed rather than aggregated. Furthermore, the incorporation of Pt SA causes distortion in the crystalline structure of both CeO_2_ and CoP, evident from Figure 3c, where a notable positive shift is observed in the CoP (011) and CeO_2_ (111) peaks compared to those of CoP@CNTs/CeO_2_–CoP@NCs, which is attributed to the compressive effect induced by the larger atomic radius of Pt atoms occupying vacancies within the CeO_2_–CoP framework.^[^ ^31^, ^32^ ^]^ This structural change leads to strong metal‐support interactions between Pt and the CeO_2_–CoP heterostructure, which not only stabilizes the Pt SA dispersion but also modifies the electronic structure of the Pt–CoP@CNTs/CeO_2_–CoP@NCs catalyst. As a result, this adjustment yields a highly reactive surface with abundant active sites and facilitates hetero‐charge transfer, ultimately benefiting catalytic performance.^[^ ^32^ ^]^ The BET surface area and pore properties of the Pt–CoP@CNTs/CeO_2_–CoP@NCs and CoP@CNTs/CeO_2_–CoP@NCs catalysts, as shown in Figure 3d, indicate that the specific surface areas are 131.2 and 116.7 m^2^ g^−1^, respectively. Moreover, while CoP@CNTs/CeO_2_–CoP@NCs demonstrates a pore size distribution in the 2–4 and 40–90 nm regions, Pt–CoP@CNTs/CeO_2_–CoP@NCs exhibits a slightly higher proportion in the 2–4 nm range and a marked decrease in the 40–90 nm range (inset of Figure 3d). This shift can be ascribed to the presence of Pt SAs, which partially obstruct larger mesopores or macropores channels, thereby decreasing the overall void volume by occupying defects or surface voids within the CeO_2_–CoP heterostructure. This process results in a reduction of larger pores in the distribution and induces a shift toward predominantly smaller microporous features.^[^ ^33^, ^34^ ^]^ The surface chemical states of Pt–CoP@CNTs/CeO_2_–CoP@NCs, CoP@CNTs/CeO_2_–CoP@NCs, and Co_3_O_4_@CNTs/CeCoO_x_@NCs catalysts were examined using XPS analysis at Korea Basic Science Institute (KBSI) in Jeonju (Figure S12, Supporting Information).The Ce 3d spectrum of Pt–CoP@CNTs/CeO_2_–CoP@NCs (Figure 3e) displays four peaks corresponding to Ce^3+^ (V_0_, V', U_0_, U') at 881.8, 886.1, 899.7, and 904.8 eV, along with six distinct peaks for Ce^4+^ (V, V″, V″, U, U″, U″) at 883.3, 887.7, 899.0, 901.7, 907.6, and 917.9 eV, which verifies the presence of both Ce^3+^ and Ce^4+^.^[^ ^35^ ^]^ A positive shift in the binding energy of Ce 3d relative to CoP@CNTs/CeO_2_–CoP@NCs indicates charge transfer from CeO_2_–CoP to Pt, thereby increasing the electron density at the d‐band center of Pt, which is beneficial for accelerating HER kinetics.^[^ ^35^, ^36^ ^]^ The Co 2p spectrum of Pt–CoP@CNTs/CeO_2_–CoP@NCs (Figure 3f) is resolved into four doublets: Co─P (778.7 and 793.2 eV), Co^3+^ (782.2 and 798.2 eV), Co^2+^ (784.9 and 799.7 eV), and satellite features (788.6 and 803.8 eV).^[^ ^10^ ^]^ The O1s XPS spectrum (Figure 3g) identifies lattice oxygen associated with the metal–oxygen bond (O_α_, 531.2 eV), oxygen vacancies (O_β_, 532.1 eV),^[^ ^37^, ^38^ ^]^ and adsorbed oxygen or water molecules (O_γ_, 533.5 eV).^[^ ^39^ ^]^ The observed positive shift in the O 1s peak for Pt–CoP@CNTs/CeO_2_–CoP@NCs or CoP@CNTs/CeO_2_–CoP@NCs demonstrates the effect of Pt–driven formation of Pt─O bonds or the generation of metal phosphides.^[^ ^40^ ^]^ The N 1s spectrum (Figure 3h) features peaks that correspond to pyridinic N (398.4 eV), pyrrolic N (401.2 eV), and graphitic N (405.2 eV), illustrating nitrogen incorporation into the carbon lattice, which results in increased defect density and active sites, thereby enhancing both conductivity and reactivity.^[^ ^23^ ^]^ The C 1s spectrum (Figure 3i) displays peaks at 284.8, 285.8, and 289.7 eV, attributed to C─C, C─O─C, and C═O bonds, respectively.^[^ ^41^ ^]^ The P 2p spectrum (Figure 3j) presents two peaks at 130.0 and 130.9 eV, corresponding to P 2p_3/2_ and P 2p_1/2_, respectively, characteristic of phosphide species, along with an additional peak at 134.1 eV assigned to the P ─ O bond.^[^ ^42^ ^]^ Interestingly, since the impregnation process is used for integrating Pt onto CoP@CNTs/CeO_2_–CoP@NCs via cation or anion‐vacancy defects on CeO_2_ or CoP surfaces, such vacancy defects having both atomic scale or cluster scale are very unstable and reactive to reduce Pt^4+^ into Pt SAs without the necessity of an additional reducing agent. This resulted in most Pt SAs are stably confined in crystalline planes via the formation of stable bonds of Pt─O or Pt─Ce/Co bonds, consistent with Pt^δ+^ characteristics owning a peak doublet appearing at binding energy of 72.6 and 75.9 eV (Figure 3k).^[^ ^43^ ^]^ Meanwhile some Pt SAs can be mobile and create individual Pt clusters, which are extremely small and low content to be detected by XRD analysis; however, their surface chemistry can be revealed by high‐resolution XPS Pt4f spectrum with the presence of Pt^°^ binding energy at 71.5 and 74.8 eV.

**Figure 4:**
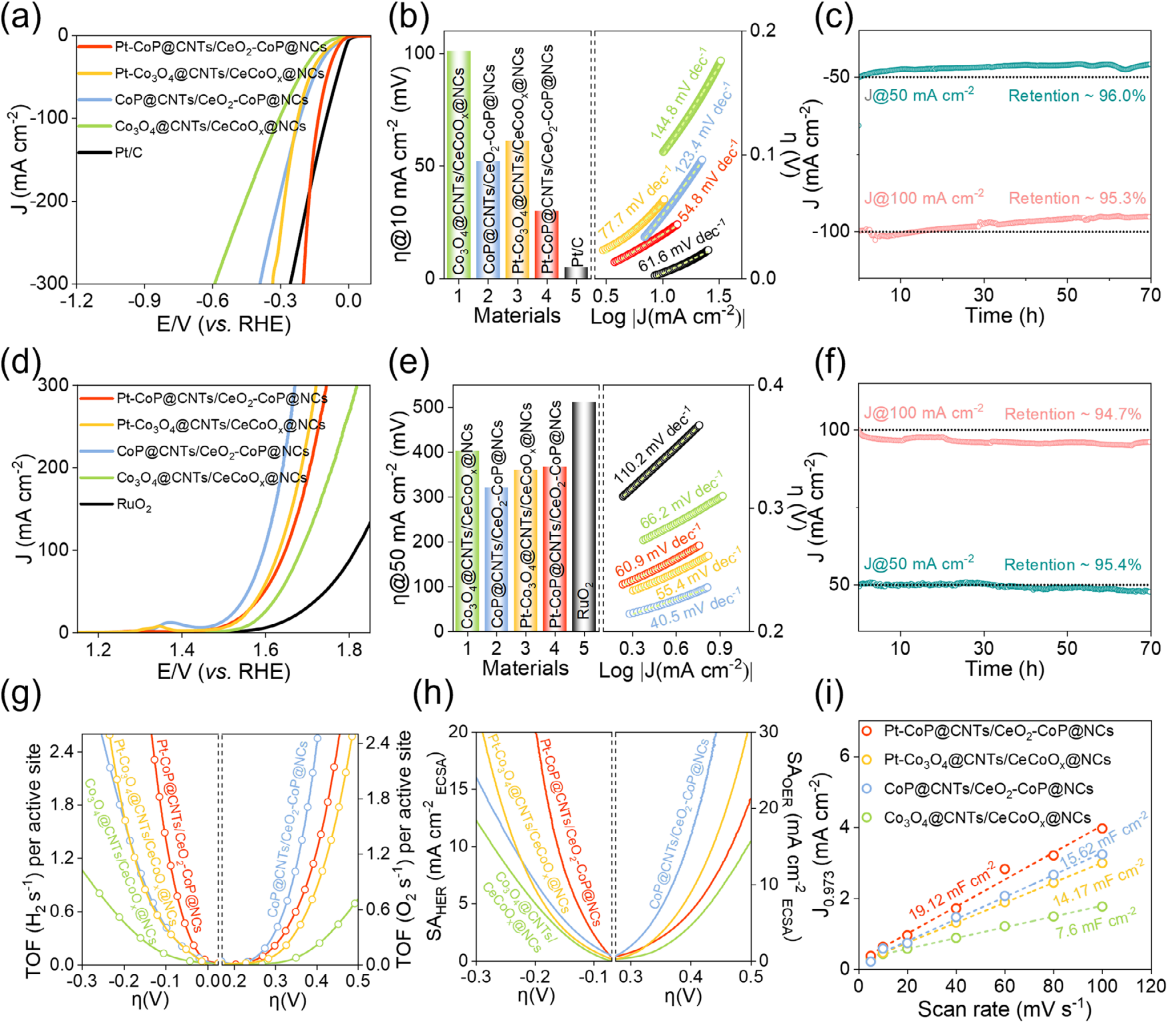
a) LSV curves, and b) the corresponding η_10_ values and Tafel slopes of the various catalysts for HER in 1.0 m KOH solution, c) durability evaluation of Pt–CoP@CNTs/CeO_2_–CoP@NCs for HER at different initial current responses; d) LSV curves, and e) the respective η_50_ values and Tafel slopes of different catalysts for OER in 1.0 m KOH solution, f) durability study of CoP@CNTs/CeO_2_–CoP@NCs for OER at different initial current responses; g) TOF, h) SA, and i) C_dl_ plots for the synthesized materials.

The catalytic efficiencies of the developed catalysts for HER and OER were assessed in a three‐electrode setup utilizing linear sweep voltammetry (LSV) in 1.0 m KOH electrolyte at 25 °C. The iR‐corrected LSV profile for Pt–CoP@CNTs/CeO_2_–CoP@NCs reveals a markedly superior HER catalytic activity, exceeding the performance of other prepared catalysts and even surpassing commercial Pt/C at current densities higher than 184 mA cm^−2^ (**Figure** **4**a). As detailed in Figure 4b, the Pt–CoP@CNTs/CeO_2_–CoP@NCs catalyst requires an overpotential (η) of just 30 mV to attain a current density of 10 mA·cm^−2^, which is 22, 31, and 71 mV lower than those for CoP@CNTs/CeO_2_–CoP@NCs, Pt–Co_3_O_4_@CNTs/CeCoO_x_@NCs, and Co_3_O_4_@CNTs/CeCoO_x_@NCs, respectively. This value of η is also lower than many recently published HER catalysts (Table S1, Supporting Information). The Tafel slope, an important criterion to characterize HER kinetics and elucidate the reaction pathway, was measured as 54.8 mV dec^−1^ for Pt–CoP@CNTs/CeO_2_–CoP@NCs, considerably smaller compared to those of Pt/C (61.6 mV dec^−1^), Pt–Co_3_O_4_@CNTs/CeCoO_x_@NCs (77.7 mV dec^−1^), CoP@CNTs/CeO_2_–CoP@NCs (123.4 mV dec^−1^), and Co_3_O_4_@CNTs/CeCoO_x_@NCs (144.8 mV dec^−1^) observed Tafel slope lies between those indicative of the Heyrovsky step (40 mV dec^−1^) and the Volmer step (120 mV dec^−1^), demonstrating enhanced electron‐transfer kinetics for Pt–CoP@CNTs/CeO_2_–CoP@NCs via the Volmer–Heyrovsky mechanism, wherein the Volmer step is the rate‐determining step (RDS).^[^ ^44^ ^]^

Stability is a critical requirement for the practical deployment of catalysts, which was evaluated through an extended chronoamperometric test at current densities of 50 and 100 mA cm^−2^. As presented in Figure 4c, Pt–CoP@CNTs/CeO_2_–CoP@NCs demonstrated remarkable durability by retaining 96.0% and 95.3% of its catalytic activity after ≈70 h of continuous operation. Furthermore, prolonged cyclic voltammetry (CV) cycling evaluations (Figure S13, Supporting Information) revealed a negligible *η*_100_ change of only 25 mV after 30 000 cycles, underscoring its superior stability. The high durability of Pt–CoP@CNTs/CeO_2_–CoP@NCs is mainly ascribed to its exceptional structural integrity, as verified by post‐HER FE‐SEM, EDS, XRD, and XPS analyses. FE‐SEM imaging (Figure S14, Supporting Information) indicated that the microcube structures integrated with hierarchical CNTs remained well‐preserved after HER. EDS confirmed that all expected elements were maintained. Moreover, the XRD profile (Figure S15, Supporting Information) demonstrated that the material post‐HER retained its characteristic diffraction peaks corresponding to the original phases, and no new crystalline phases appeared. The decrease in peak intensity and slight shifts in diffraction patterns pointed to minor leaching of active species and structural distortion, likely resulting from the infiltration of intermediates or electrolytes during HER. The XPS spectra shown in Figure S16 (Supporting Information) verified the existence of Ce, Co, Pt, P, O, C, and N binding energies in the post‐HER sample. However, notable changes in surface chemistry were observed, such as the disappearance of Co─P peaks in the Co 2p spectrum and the reduction in intensity of the P 2p_1/2_ and P 2p_3/2_ components in the P 2p spectrum, which suggests surface oxidation occurred during the HER process.

The anodic OER characteristics of the synthesized catalyst materials, shown in Figure 4d, reveal that CoP@CNTs/CeO_2_–CoP@NCs delivers markedly superior catalytic efficiency compared to other examined samples. Interestingly, a small peak appearing ≈1.3 V (vs RHE) in the OER LSV of CoP@CNTs/CeO_2_–CoP@NCs attributes to the oxidation of transition metal pieces, such as cobalt, to create a thin surface layer of high‐valence Co oxides/hydroxides, which is highly active to promote the subsequent OER process. Importantly, this catalyst reaches a current density of 50 mA cm^−2^ at an η_50_ value of just 320 mV (Figure 4e), which is lower than those required for Pt–Co_3_O_4_@CNTs/CeCoO_x_@NCs (360 mV), Pt–CoP@CNTs/CeO_2_–CoP@NCs (367 mV), Co_3_O_4_@CNTs/CeCoO_x_@NCs (403 mV), and the benchmark RuO_2_ (511 mV). This *η*_50_ performance also exceeds recently reported OER catalysts (Table S2, Supporting Information). Further kinetic analysis shows that CoP@CNTs/CeO_2_–CoP@NCs features a Tafel slope of 40.5 mV dec^−1^, which is noticeably lower than that of Pt–Co_3_O_4_@CNTs/CeCoO_x_@NCs (55.4 mV dec^−1^), Pt–CoP@CNTs/CeO_2_–CoP@NCs (60.9 mV dec^−1^), Co_3_O_4_@CNTs/CeCoO_x_@NCs (66.2 mV dec^−1^), and RuO_2_ (110.2 mV dec^−1^), indicating accelerated OER reaction kinetics. The operational reliability of CoP@CNTs/CeO_2_–CoP@NCs during OER was demonstrated via extended chronoamperometric testing at 50 and 100 mA cm^−2^ (Figure 4f). The catalyst maintained excellent performance, retaining 95.4% and 94.7% of its initial activity at these current densities after 70 h of uninterrupted operation. Additional continuous CV cycling tests confirmed the stability of CoP@CNTs/CeO_2_–CoP@NCs, as shown in Figure S17 (Supporting Information), which shows only a minor *η*_100_ increase of 20 mV after 30 000 CV cycles. The exceptional durability of CoP@CNTs/CeO_2_–CoP@NCs is mainly ascribed to its robust structure, as additionally verified by FE‐SEM, EDS, XRD, and XPS analyses. FE‐SEM images in Figure S18 (Supporting Information) confirm the retention of microcube morphology embedded with hierarchical CNTs after OER, and EDS results show all constituent elements remain present. Furthermore, Figure S19 (Supporting Information) presents the post‐OER XRD pattern, revealing preserved diffraction peaks of the original phases and new crystalline peaks of Co(OH)_2_. This phase transformation is likely caused by partial removal of the phosphorus component from CoP, leading to destabilization and generation of Co(OH)_2_ during the OER process. The slight reduction in peak intensities and minor shifts in the XRD pattern suggest partial loss of active species and structural changes due to intermediate or electrolyte interactions. In agreement with observations during HER, XPS characterization of the post‐OER samples demonstrates surface oxidation, as shown by the disappearance of the Co─P bond and the notable reduction of the P 2p_1/2_ and P 2p_3/2_ peaks within the P 2p spectrum (Figure S20, Supporting Information). To examine the intrinsic catalytic activities of the synthesized materials, turnover frequency (TOF) was assessed using the CV method in PBS solution (Figure S21, Supporting Information), indicating that Pt–CoP@CNTs/CeO_2_–CoP@NCs for HER and CoP@CNTs/CeO_2_–CoP@NCs for OER demonstrate the highest activity among the tested catalysts. For HER, Pt–CoP@CNTs/CeO_2_–CoP@NCs attains a TOF of 1.5 s^−1^ at an η of 0.1 V, which is 3.4, 3.75, and 13.6 times greater than those of CoP@CNTs/CeO_2_–CoP@NCs (0.44 s^−1^), Pt–Co_3_O_4_@CNTs/CeCoO_x_@NCs (0.40 s^−1^), and Co_3_O_4_@CNTs/CeCoO_x_@NCs (0.11 s^−1^), respectively. For OER, CoP@CNTs/CeO_2_–CoP@NCs achieves a TOF value of 2.4 s^−1^ at η = 0.4 V, which is higher than those of Pt–CoP@CNTs/CeO_2_–CoP@NCs (1.2 s^−1^), Pt–Co_3_O_4_@CNTs/CeCoO_x_@NCs (0.8 s^−1^), and Co_3_O_4_@CNTs/CeCoO_x_@NCs (0.2 s^−1^), respectively (Figure 4g). ECSA‐normalized LSV results further confirm the higher SA activity of Pt–CoP@CNTs/CeO_2_–CoP@NCs for HER and CoP@CNTs/CeO_2_–CoP@NCs for OER. During HER, Pt–CoP@CNTs/CeO_2_–CoP@NCs exhibits an SA of 20.0 mA cm^−2^ at η = 0.2 V, outperforming CoP@CNTs/CeO_2_–CoP@NCs (7.1 mA cm^−2^), Pt–Co_3_O_4_@CNTs/CeCoO_x_@NCs (6.5 mA cm^−2^), and Co_3_O_4_@CNTs/CeCoO_x_@NCs (5.1 mA cm^−2^).Similarly, for OER, CoP@CNTs/CeO_2_–CoP@NCs achieves an SA of 16.6 mA cm^−2^ at η = 0.4 V, which is significantly higher than those of Pt–Co_3_O_4_@CNTs/CeCoO_x_@NCs (8.9 mA cm^−2^), Pt–CoP@CNTs/CeO_2_–CoP@NCs (6.5 mA cm^−2^), and Co_3_O_4_@CNTs/CeCoO_x_@NCs (4.6 mA cm^−2^) (Figure 4h). Additionally, the Nyquist plots presented in Figure S22 (Supporting Information) demonstrate that Pt–CoP@CNTs/CeO_2_–CoP@NCs and CoP@CNTs/CeO_2_–CoP@NCs exhibit remarkably smaller semicircle diameters in the high‐frequency region, reflecting lower charge transfer resistance (R_ct_) values of 0.45 Ω and 0.50 Ω, respectively, compared to Pt–Co_3_O_4_@CNTs/CeCoO_x_@NCs (0.69 Ω) and Co_3_O_4_@CNTs/CeCoO_x_@NCs (0.94 Ω), which verifies their enhanced charge transfer efficiency conducive to improved catalytic activity. Additionally, Figure 4i shows the electrochemical double‐layer capacitance (*C*_dl_) which serves as an indicator of the electrochemically active surface area (ECSA),^[^ ^45^ ^]^ for the synthesized catalysts. The *C*_dl_ values were determined by recording CV scans at different scan rates within the non‐faradaic region (Figure S23, Supporting Information). The *C*_dl_ value for Pt–CoP@CNTs/CeO_2_–CoP@NCs is 19.12 mF cm^−2^, which is 1.22, 1.35, and 2.52 times greater than those of CoP@CNTs/CeO_2_–CoP@NCs, Pt–Co_3_O_4_@CNTs/CeCoO_x_@NCs, and Co_3_O_4_@CNTs/CeCoO_x_@NCs, respectively. Employing the equation ECSA = *C*_dl_/*C*_s_, where C_s_ (the double‐layer capacitance of pure Ni foam in 1 m KOH) is 1.375 mF cm^−2^ (Figure S24, Supporting Information), the ECSA values of Pt–CoP@CNTs/CeO_2_–CoP@NCs, CoP@CNTs/CeO_2_–CoP@NCs, Pt–Co_3_O_4_@CNTs/CeCoO_x_@NCs, and Co_3_O_4_@CNTs/CeCoO_x_@NCs were calculated to be 13.91, 11.36, 10.31, and 5.53 cm^2^, respectively. The presence of multiple interfaces and the resulting electronic structure changes in Pt–CoP@CNTs/CeO_2_–CoP@NCs and CoP@CNTs/CeO_2_–CoP@NCs lead to improved wettability, as indicated by water contact angle (WCA) measurements (Figure S25, Supporting Information). Enhanced wettability notably facilitates adsorption and mass transfer during electrochemical reactions. Collectively, these findings confirm the superior intrinsic catalytic activities of Pt–CoP@CNTs/CeO_2_–CoP@NCs for HER and CoP@CNTs/CeO_2_–CoP@NCs for OER in a 1 m KOH solution.

**Figure 5:**
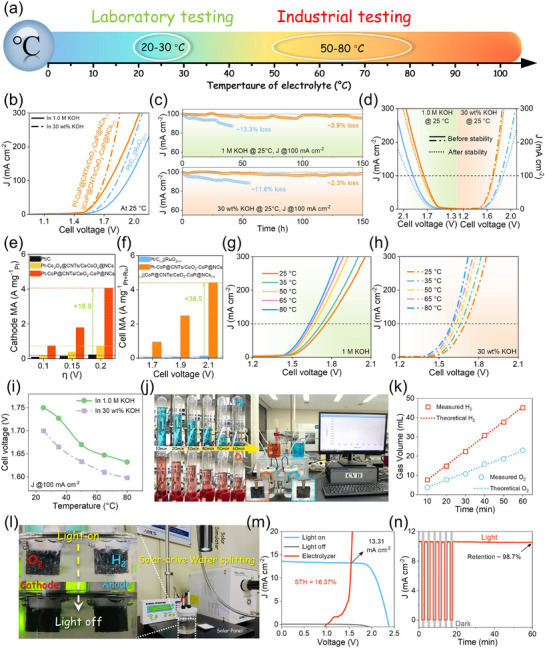
Water electrolysis evaluated under simulated industrial conditions. a) Comparison of temperature conditions used in laboratory versus industrial testing; b) LSV curves, c) chronoamperometric profiles, and d) LSV curves recorded before and after stability assessments for cells constructed from Pt–CoP@CNTs/CeO_2_–CoP@NCs_(−)_||CoP@CNTs/CeO_2_–CoP@NCs_(+)_ and Pt/C_(−)_||RuO_2(+)_ in KOH solutions of different concentrations (1 m and 30 wt.%) at 25 °C; e) Comparison of cathode mass activity for Pt–CoP@CNTs/CeO_2_–CoP@NCs, Pt–Co_3_O_4_@CNTs/CeCoO_x_@NCs, and Pt/C in HER processes; f) Cell mass activity comparison between Pt–CoP@CNTs/CeO_2_–CoP@NCs_(−)_||CoP@CNTs/CeO_2_–CoP@NCs_(+)_ and Pt/C_(−)_||RuO_2(+)_ cells; g) LSV profiles of Pt–CoP@CNTs/CeO_2_–CoP@NCs_(−)_||CoP@CNTs/CeO_2_–CoP@NCs_(+)_ in 1 m KOH and h) 30 wt.% KOH at temperatures ranging from 25 to 80 °C; i) Cell voltage at 100 mA cm^−2^ for Pt–CoP@CNTs/CeO_2_–CoP@NCs_(−)_||CoP@CNTs/CeO_2_–CoP@NCs_(+)_ across varying temperatures; j) Photographs of Pt–CoP@CNTs/CeO_2_–CoP@NCs_(−)_||CoP@CNTs/CeO_2_–CoP@NCs_(+)_ cell illustrating H_2_ and O_2_ bubble production, and k) the corresponding Faradaic efficiency; l) Configuration of the solar‐driven water‐splitting system; m) *J*–*V* curve of the solar cell along with the LSV curve of the water‐splitting device; n) *J*–*t* plot of the solar‐driven water‐splitting system under cyclic solar irradiation (on/off operation).

Although alkalinity and temperature play a crucial role in determining the performance of water electrolysis devices, there has been a lack of comprehensive studies that systematically investigate these influences. These parameters are vital for providing substantial evidence to assess their feasibility for large‐scale industrial applications. To address this shortcoming, an electrolyzer was fabricated employing Pt–CoP@CNTs/CeO_2_–CoP@NCs as the cathode and CoP@CNTs/CeO_2_–CoP@NCs as the anode (abbreviated as Pt–CoP@CNTs/CeO_2_–CoP@NCs_(−)_||CoP@CNTs/CeO_2_–CoP@NCs_(+)_), and the overall water splitting performance was evaluated in 1 m and 30 wt.% KOH electrolytes over a range of temperatures. **Figure** **5**a presents a schematic illustration comparing the water electrolysis processes at laboratory and industrial temperatures. As depicted in Figure 5b, the Pt–CoP@CNTs/CeO_2_–CoP@NCs_(−)_||CoP@CNTs/CeO_2_–CoP@NCs_(+)_ achieves a current density of 10 mA cm^−2^ at cell voltages of 1.52 and 1.53 V in 30 wt.% and 1.0 m KOH, respectively, which are considerably lower than those required for the Pt/C_(−)_||RuO_2(+)_ system and are among the top‐reported values for contemporary candidates in alkaline environments (Table S3, Supporting Information). The durability assessment of Pt–CoP@CNTs/CeO_2_–CoP@NCs_(−)_||CoP@CNTs/CeO_2_–CoP@NCs_(+)_ and Pt/C_(−)_||RuO_2(+)_ was carried out at 100 mA·cm^−2^, 25 °C in both 1.0 m and 30 wt.% KOH electrolytes (Figure 5c). The Pt–CoP@CNTs/CeO_2_–CoP@NCs_(−)_||CoP@CNTs/CeO_2_–CoP@NCs_(+)_ demonstrated stable performance over 150 h, with only a slight potential drop of ≈3.9% in 1 m KOH and ≈2.3% in 30 wt.% KOH, while Pt/C_(−)_||RuO_2(+)_ underwent rapid reduction in activity after ≈50 h under the same testing parameters. The qualification of dissolved metal ions from the catalyst into the electrolyte by ICP‐MS after the stability test indicates a concentration of 1.104 ppm for Co ions and 1.072 ppm for Ce ions in 1 m KOH while a concentration of 9.990 ppm for Co ions and 9.993 ppm for Ce ions is found in 30 wt.% KOH media. There is no Pt concentration is detected because either the dissolved Pt content is below the instrument detection limit or strong immobilization of Pt SAs in the CoP@CNTs/CeO_2_–CoP@NCs. LSV results captured before and after the cycling tests revealed minor overpotential increases (Δη) of 0.034 and 0.015 V for the Pt–CoP@CNTs/CeO_2_–CoP@NCs_(−)_||CoP@CNTs/CeO_2_–CoP@NCs_(+)_ configuration at 100 mA cm^−2^ in 1 m and 30 wt.% KOH, respectively. By contrast, Pt/C_(−)_||RuO_2(+)_ displayed much higher Δη values of 0.080 and 0.033 V (Figure 5d). These findings underscore the exceptional durability of Pt–CoP@CNTs/CeO_2_–CoP@NCs_(−)_||CoP@CNTs/CeO_2_–CoP@NCs_(+)_ during extended water splitting operations. This achievement may be due to the fact that the highly porous architecture of CoP@CNTs/CeO_2_–CoP@NCs could produce multiple channels for electrolyte penetration and gas diffusion. In addition, the formation of CeO_2_/CoP heterostructures significantly enhances interfacial electronic interactions and accelerates charge transfer, thereby reinforcing both the catalytic activity and structural stability of the material. Furthermore, the encapsulation of CoP NPs within conductive CNTs provides a protective and supportive matrix, which effectively mitigates direct exposure to the electrolyte and suppresses the leaching of active species from the active surface into solution. Thus, these synergistic effects ensures remarkable electrochemical stability of the catalyst during water splitting operation. To assess the economic viability of the Pt–CoP@CNTs/CeO_2_–CoP@NCs_(−)_||CoP@CNTs/CeO_2_–CoP@NCs_(+)_ system, the mass activity (MA) for both the cathode and the complete cell was systematically investigated. Figures 5e and S26 (Supporting Information) demonstrate that the cathode MA of Pt–CoP@CNTs/CeO_2_–CoP@NCs is markedly superior to those of Pt–Co_3_O_4_@CNTs/CeCoO_x_@NCs and Pt/C. Specifically, the MA of Pt–CoP@CNTs/CeO_2_–CoP@NCs at η of 0.2 V was 18.9 times higher compared to Pt/C. Furthermore, the cell MA of the Pt–CoP@CNTs/CeO_2_–CoP@NCs_(−)_||CoP@CNTs/CeO_2_–CoP@NCs_(+)_ cell was 38.5‐fold greater than that of the commercial Pt–C_(−)_//RuO_2(+)_ cell at 2.1 V (Figures 5f; S27, Supporting Information). Typical operating temperatures for industrial water electrolysis systems range from 50 to 80 °C, facilitating a reduction in the overall voltage necessary for water splitting.^[^ ^46^, ^47^ ^]^ The electrochemical characteristics of water splitting electrolyzers should be assessed at these industrially relevant temperatures to better reconcile laboratory results with practical application.

The overall water splitting polarization curves of Pt–CoP@CNTs/CeO_2_–CoP@NCs_(−)_||CoP@CNTs/CeO_2_–CoP@NCs_(+)_ were recorded at temperatures ranging from 25 to 80 °C in 1 m and 30 wt.% KOH electrolytes. As shown in Figure 5g,h, increasing the temperature of the alkaline electrolyte progressively lowers the voltage required for efficient water splitting. This effect results from enhanced mass transport and ion mobility due to decreased activation energy, along with the accelerated release of gas bubbles.^[^ ^48^ ^]^ At a current density of 100 mA cm^−2^, the water splitting voltages for Pt–CoP@CNTs/CeO_2_–CoP@NCs_(−)_||CoP@CNTs/CeO_2_–CoP@NCs_(+)_ in 30 wt.% KOH remain consistently lower compared to 1 m KOH across a range of temperatures (Figure 5i), underscoring the suitability of Pt–CoP@CNTs/CeO_2_–CoP@NCs_(−)_||CoP@CNTs/CeO_2_–CoP@NCs_(+)_ for large‐scale water electrolysis. Furthermore, the Faradaic efficiency (η_F_) was determined from the total charge passed through the electrolyzer cell and the volume of evolved gas over 60 min of continuous operation at 100 mA cm^−2^, with Pt–CoP@CNTs/CeO_2_–CoP@NCs as the cathode and CoP@CNTs/CeO_2_–CoP@NCs as the anode (Figure 5j). The H_2_ and O_2_ evolution, monitored every 10 min by the drainage method, exhibited close agreement with theoretical calculations, demonstrating promising η_F_ values of ≈98.9% for H_2_ and 98.3% for O_2_ production, which reflects the high catalytic selectivity of the system (Figure 5k). To demonstrate the feasibility of full green hydrogen production by water splitting, a lab‐scale solar‐powered Pt–CoP@CNTs/CeO_2_–CoP@NCs_(−)_||CoP@CNTs/CeO_2_–CoP@NCs_(+)_ electrolyzer cell was constructed, as depicted in Figure 5l. The cell's operation was assessed under the switching cycles of simulated solar irradiation. As presented in Figure 5l, when the solar panel is exposed to simulated sunlight, a substantial evolution of gas bubbles is observed at the electrode surface, verifying the effective water splitting enabled by solar energy. The intersection of the *J*–*V* curve for the solar cell (AM 1.5 G simulated sunlight) and the LSV profile of the electrolyzer identifies a current density of 13.31 mA cm^−2^, yielding a solar‐to‐hydrogen (STH) conversion efficiency of 16.37% (Figure 5m). Additionally, long‐term performance evaluation under alternating light‐on and light‐off cycles (Figure 5n) demonstrates a rapid current response, highlighting the suitability of the Pt–CoP@CNTs/CeO_2_–CoP@NCs_(−)_||CoP@CNTs/CeO_2_–CoP@NCs_(+)_−based electrolyzer for practical solar‐driven water splitting to generate green hydrogen.

**Figure 6:**
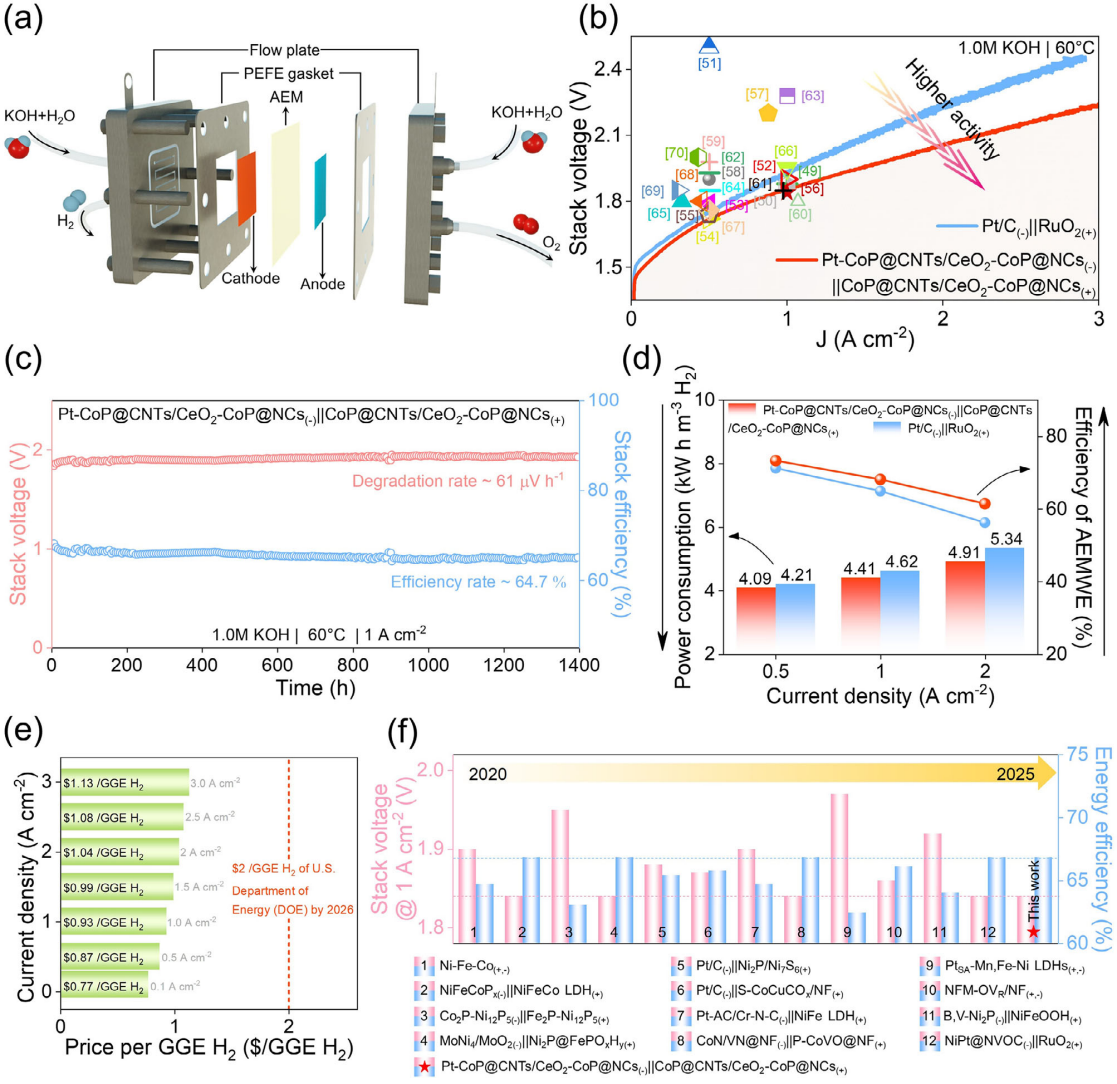
a) Schematic of the AEMWE stack performance testing platform; b) LSV curves for the Pt–CoP@CNTs/CeO_2_–CoP@NCs_(−)_||CoP@CNTs/CeO_2_–CoP@NCs_(+)_ stack; c) Long‐term durability evaluation of the Pt–CoP@CNTs/CeO_2_–CoP@NCs_(−)_||CoP@CNTs/CeO_2_–CoP@NCs_(+)_ AEMWE stack at 1 A cm^−2^ over 1400 h under simulated industrial conditions; d) Comparative power consumption and efficiency for Pt–CoP@CNTs/CeO_2_–CoP@NCs_(−)_||CoP@CNTs/CeO_2_–CoP@NCs_(+)_ and Pt/C_(−)_||RuO_2(+)_ AEMWE stacks; e) Projected production cost of 1.0 kg hydrogen by AEM electrolysis at different current densities in 1.0 m KOH; f) Compilation of recent data on stack voltage and energy efficiency for AEMWE.

To assess the practical applicability of the catalyst under simulated industrial conditions, the Pt–CoP@CNTs/CeO_2_–CoP@NCs_(−)_||CoP@CNTs/CeO_2_–CoP@NCs_(+)_ was assembled into an anion exchange membrane water electrolyzer (AEMWE) stack and subjected to performance and durability evaluations using an electrolyzer tester (**Figure** **6**a). The LSV curves (without IR correction) for the Pt–CoP@CNTs/CeO_2_–CoP@NCs_(−)_||CoP@CNTs/CeO_2_–CoP@NCs_(+)_−based AEMWE stack revealed current densities of 0.5, 1.0, and 2.0 A cm^−2^ at stack voltages of 1.71, 1.84, and 2.04 V, respectively (Figure 6b), with testing conducted in 1.0 m KOH at 60 °C. These voltages are substantially lower than those recorded for the Pt/C_(−)_||RuO_2(+)_−based AEMWE stack (1.76, 1.93, and 2.23 V), and demonstrate strong competitiveness compared with the latest reported AEMWE stacks (Table S4, Supporting Information).^[^ ^49^, ^50^, ^51^, ^52^, ^53^, ^54^, ^55^, ^56^, ^57^, ^58^, ^59^, ^60^, ^61^, ^62^, ^63^, ^64^, ^65^, ^66^, ^67^, ^68^, ^69^, ^70^ ^]^

The durability of the Pt–CoP@CNTs/CeO_2_–CoP@NCs_(−)_||CoP@CNTs/CeO_2_–CoP@NCs_(+)_ stack was evaluated at a high current density of 1 A cm^−2^ (Figure 6c). Remarkably, the stack operated stably and exhibited a degradation rate of only 61 µV h^−1^ even after 1000 h of continuous testing. As a result, the cell efficiency of the Pt–CoP@CNTs/CeO_2_–CoP@NCs_(−)_||CoP@CNTs/CeO_2_–CoP@NCs_(+)_ AEMWE declined by just 3.4%, from 68.1% to 64.7%, after 1000 h of operation. Furthermore, the stack achieved high energy efficiencies of 66.8% and 60.0% at current densities of 1.0 and 2.0 A cm^−2^, respectively, corresponding to energy consumptions of 48.95 and 54.52 kWh kg^−1^ H_2_. To more comprehensively assess its practical viability, the power consumption and AEMWE efficiency of the Pt–CoP@CNTs/CeO_2_–CoP@NCs_(−)_||CoP@CNTs/CeO_2_–CoP@NCs_(+)_ stack were compared with those of the Pt/C_(−)_||RuO_2(+)_ stack at different current densities (Figure 6d). The Pt–CoP@CNTs/CeO_2_–CoP@NCs_(−)_||CoP@CNTs/CeO_2_–CoP@NCs_(+)_ catalyst exhibits a low energy consumption of 4.91 kW h m^−3^ and a high AEMWE efficiency of 61.4% at 2 A cm^−2^, surpassing the performance of the Pt/C_(−)_||RuO_2(+)_ stack, which shows values of 5.34 kW h m^−3^ and 56.2%, respectively. A crucial metric for evaluating the economic performance of the AEM electrolyzer, the cost per gasoline gallon equivalent (GGE) of H_2_, is as low as $0.77, as indicated in Figure 6e. This value is significantly below the U.S. Department of Energy's (DOE) 2026 target of $2.00.^[^ ^41^ ^]^ Additionally, the stack achieved high energy efficiencies of 66.8% and 60.0% at current densities of 1.0 and 2.0 A cm^−2^, respectively, corresponding to energy usage of 48.95 and 54.52 kWh kg^−1^ H_2_. Moreover, the AEM water electrolyzer demonstrates excellent performance at the high current density of 1.0 A cm^−2^ and remains competitive among recent state‐of‐the‐art studies (Figure 6f; Table S4, Supporting Information). These findings highlight the outstanding performance and significant practical potential of the Pt–CoP@CNTs/CeO_2_–CoP@NCs_(−)_||CoP@CNTs/CeO_2_–CoP@NCs_(+)_ stack for industrial‐scale water‐splitting applications. To the best of our knowledge, this AEM water electrolyzer demonstrates some of the highest large‐current‐density (1.0 A cm^−2^) alkaline water electrolysis performance reported to date.

## 3 Conclusion

In this study, we established a novel catalyst engineering approach based on the self‐templated growth of CeCo–MOF, which was subsequently subjected to selective phosphidization and Pt single‐atom implantation to synthesize highly efficient CoP@CNTs/CeO_2_–CoP@NCs and Pt–CoP@CNTs/CeO_2_–CoP@NCs, demonstrating strong catalytic performance for OER and HER, respectively. Consequently, the two‐electrode electrolyzer Pt–CoP@CNTs/CeO_2_–CoP@NCs_(−)_||CoP@CNTs/CeO_2_–CoP@NCs_(+)_ exhibited a cell voltage of 1.52 V in 30 wt.% KOH and 1.53 V in 1.0 M KOH, outperforming the commercial Pt/C_(−)_||RuO_2(+)_ system. The AEMWE stack constructed from Pt–CoP@CNTs/CeO_2_–CoP@NCs_(−)_||CoP@CNTs/CeO_2_–CoP@NCs_(+)_ achieved a stack voltage of only 1.71/1.84/2.04 V to reach current densities of 0.5/1/2 A cm^−2^ in 1.0 M KOH at 60 °C and maintained stable operation for 1400 h with a minimal degradation rate of only 61 µV h^−1^, demonstrating performance on par with leading outcomes reported in recent AEMWE research. These findings highlight the significant promise of the developed catalyst systems for practical, efficient, and sustainable water‐splitting applications.

## 4 Experimental Section

### Synthesis of CeCo–MOF Material

Typically, 580 mg of Co(NO_3_)_2_·6H_2_O, 175 mg of Ce(NO_3_)_3_⋅6H_2_O, and 10 mg of CTAB were dissolved in 20 mL of DI water to create Solution A. Solution A was rapidly mixed with Solution B, consisting of 9.08 g of 2‐Methylimidazole dissolved in 140 mL of DI water, and the mixture was stirred for 30 min at room‐temperature. The resulting precipitate was separated by centrifugation, washed thoroughly with DI water at least six times, and dried in a vacuum oven at 60 °C for 12 h.

### Synthesis of Co_3_O_4_@CNTs/CeCoO_X_@NCs and CoP@CNTs/CeO_2_–CoP@NCs Material

The CeCo–MOF precursor was placed in a quartz boat and heated to 800 °C at a ramp rate of 5 °C min^−1^ in an Ar/H_2_ (90/10) atmosphere. This temperature was sustained for 2 h to facilitate CNT growth. The sample was then annealed in a furnace at 280 °C for 2 h (2 °C min^−1^) in air to fully convert the precursor to the oxide, resulting in Co_3_O_4_@CNTs/CeCoO_x_@NCs.

For the synthesis of CoP@CNTs/CeO_2_–CoP@NCs, 0.05 g of Co_3_O_4_@CNTs/CeCoO_x_@NCs and 0.5 g of Na_2_H_2_PO_2_·H_2_O were placed in separate quartz boats at the downstream and upstream positions of a tube furnace, respectively. The sample was subjected to a phosphidization reaction at 350 °C for 2 h (2 °C min^−1^) under an Ar flow and then cooled to room‐temperature to obtain CoP@CNTs/CeO_2_–CoP@NCs (Scheme 1).

### Synthesis of Pt–CoP@CNTs/CeO_2_–CoP@NCs Material

To synthesize Pt–CoP@CNTs/CeO_2_–CoP@NCs, 0.035 g of CoP@CNTs/CeO_2_–CoP@NCs was dispersed in DI water by sonication using an ice bath. Then, 0.3 mm of the Pt ion precursor was quickly added while stirring magnetically for 180 s to promote the incorporation of Pt single atoms into the material. The preparation of Pt–Co_3_O_4_@CNTs/CeCoO_x_@NCs was performed following this identical procedure. For comparison, control samples with varying Pt ion precursor concentrations were also synthesized and are denoted as Pt–CoP@CNTs/CeO_2_–CoP@NCs‐0.1 (0.1 mm H_2_PtCl_6_) and Pt–CoP@CNTs/CeO_2_–CoP@NCs−0.4 (0.4 mm H_2_PtCl_6_).

## Conflict of Interest

The authors declare no conflict of interest.

## Supporting information



Supporting Information

## Data Availability

The data that support the findings of this study are available from the corresponding author upon reasonable request.
