# Supplementary material for: Atomic Pt‐Promoted Hierarchical CoP@CNTs‐Bridged CeO2–CoP Heterostructure‐Based Hollow Microcubes for Water Electrolysis
Source: Small. 2025 Oct 14;21(46):e07407. doi: 10.1002/smll.202507407 (PMC12632438; doi:10.1002/smll.202507407)
Supplement: Supplementary file 1 — Supporting Information [file SMLL-21-e07407-s001.docx]

**Supporting Information**

**Atomic Pt−promoted Hierarchical CoP@CNTs−bridged CeO_2_−CoP Heterostructure-based Hollow Microcubes for Water Electrolysis**

Xue Li, Kaixuan Dong, Saleem Sidra, Do Hwan Kim, Duy Thanh Tran, Nam Hoon Kim^*^, Joong Hee Lee^*^

X Li, K Dong, D. T. Tran, N. H. Kim, J. H. Lee

Department of Nano Convergence Engineering, Jeonbuk National University, Jeonju, Jeonbuk 54896, Republic of Korea.

S. Sidra and D. H. Kim

Division of Science Education, Graduate School of Department of Energy Storage/Conversion Engineering, Jeonbuk National University Jeonju, Jeonbuk, 54896 Republic of Korea.

J. H. Lee

Carbon Composite Research Center, Department of Polymer-Nano Science and Technology, Jeonbuk National University, Jeonju, Jeonbuk 54896, Republic of Korea.

**Email:** nhk@jbnu.ac.kr and jhl@jbnu.ac.kr.

**Chemical reagents.** Cobalt(II) nitrate hexahydrate (Co(NO_3_)_2_·6H_2_O, ≥ 98.0%), cerium nitrate hexahydrate (Ce(NO_3_)_3_⋅6H_2_O, 99% trace metals basis), sodium hypophosphite monohydrate (NaH_2_PO_2_·H_2_O, 99.9%), 2-Methylimidazole (C_4_H_6_N_2_, 99 %), cetyl trimethyl ammonium bromide (CTAB, ≥99%), Nafion solution (5 wt%), Pt on graphitized carbon (20 wt% Pt), and ruthenium(IV) oxide (RuO_2_, 99.9 %), chloroplatinic acid hexahydrate (H_2_PtCl_6_·6H_2_O, ≥37.50% Pt basis) were provided by Sigma-Aldrich Co. (USA). Potassium hydroxide (KOH, 85 %), ethanol (C_2_H_5_OH, 99 %), and hydrochloric acid (HCl, 37.5 wt%) were obtained from Samchun Co. (Korea). Deionized (DI) water was filtered using an EYELA Still Ace SA-2100E1 system from Tokyo Rikakikai Co. (Japan). Nickel foam (NF) was purchased from Suzhou Yilongsheng Energy Technology Co. (China). All reagents were used as received without any further purification.

**Structural Characterizations.** Microstructure of the synthesized materials was observed using field emission scanning electron spectroscopy (FE-SEM) with a Supra 40 VP instrument (Zeiss Co., Germany), equipped with an energy-dispersive X-ray spectroscopy (EDS) detector, and by transmission electron microscopy (TEM) on a JEM-ARM200F system (JEOL Co., Japan) at the CURF. X-ray diffraction (XRD) was carried out on D/Max 2500 V/Pc (Japan) using Cu Kα radiation (λ = ∼1.54 Å) over a range of 5–80° (2° min^−1^). The Theta Probe machine (Thermo Fisher Scientific, USA) was employed to perform X-ray photoelectron spectroscopy (XPS) analyses on the surface chemistry of the materials. The specific surface area and pore volume of the materials were measured using an ASAP 2020 Plus system (Micromeritics Instrument Corp., USA). Water contact angle (WCA) measurements were conducted using a DSA25 Drop Shape Analyzer (Krüss GmbH, Germany).

**Electrochemical characterizations.** Electrochemical characterization of the prepared electrodes was carried out using a CHI 660D workstation (CH Instruments Inc., USA) equipped with a three−electrode system, where nickel foam (1 × 1 cm) acted as the working electrode, and a graphite rod and Ag/AgCl electrode served as the counter and reference electrodes, respectively. To fabricate the electrocatalyst ink, 4 mg of the synthesized catalysts were dispersed in 500 µL of anhydrous ethyl alcohol (99.9%) with 30 µL of Nafion (5 wt%), followed by sonication for 2 hours in an ice bath. The resulting ink was then coated onto nickel foam (NF) and left to dry naturally overnight. For reference, commercial catalysts such as Pt/C (20 wt% Pt loading) and RuO₂ (≥99.9 wt%) were prepared at the same volume loading as the synthesized catalysts on NF. The HER and OER activities were evaluated by performing linear sweep voltammetry (LSV) at a scan rate of 1 mV·s⁻¹ in 1.0 M KOH electrolyte until achieving overlapping curves, after which iR−compensation (100%) was applied. Electrochemical impedance spectroscopy (EIS) measurements were conducted under open−circuit potential from 10^−2^ to 10^5^ Hz with a 0.005 V amplitude. The electrochemical surface area (ECSA) was estimated by cyclic voltammetry (CV) over various scan rates (5, 10, 20, 40, 60, 80, and 100 mV s^−1^) in the non−faradaic region to obtain the double−layer capacitance (C_dl_). Catalytic durability was determined by monitoring LSV changes before and after 30000 CV cycles and by applying the chronoamperometry method. Potentials from all electrochemical tests were converted to reversible hydrogen electrode (RHE) values using the following equation:

| $E/V (vs. RHE)=E_{{Ag}/{AgCl}} +0.197+0.0591\times pH$ | (1) |
| --- | --- |

In the solar−driven water splitting process, the system includes the Pt−CoP@CNTs/CeO_2_−CoP@NCs_(−)_||CoP@CNTs/CeO_2_−CoP@NCs_(+)_ electrodes, a commercial solar cell panel (5 cm × 5 cm, active surface area: 11.55 cm^2^ ), and a HAL−320 solar simulator (Asahi Spectra Co., Ltd., Japan). The solar−to−hydrogen (STH) efficiency was calculated using the following formula:

| $STH Efficiency \left( \% \right)= \frac{\left( J \times1\cdot23 V \right)}{100 mW {cm}^{-2}} \times100$ | (2) |
| --- | --- |

Here, J denotes the measured current density at the point where the LSV response from OWS intersects with the (J−V) curve obtained from the solar cell. The minimum theoretical voltage needed to split water into hydrogen and oxygen is 1.23 V, while 100 mW cm^−2^ specifies the solar power density, representing the intensity of the incident solar energy.

**Membrane Activation, GDL Coating, Fabrication of the AEMWE Stack.** An AEMWE single-cell was assembled with a 4.0 cm^2^ device. The Sustainion (X37-50, Grade T) membrane, carbon cloth (CeTech W1S1010), and stainless-steel fiber felt (SS fiber felt, 0.45 mm) were used as the gas diffusion layers (GDLs) for the cathode and anode, respectively. In details, each membrane should be pretreated separately in 1.0 M KOH solution for at least 24 hours to ensure fully exchange of chloride ions for hydroxide ions in the membrane. The GDLs for the cathode (1.5 cm × 1.5 cm) and anode (1.75 cm × 1.75 cm) were cut precisely, washed with acetone solution, and dried in an oven. Then, the GDLs were affixed to a hotplate maintain at 80 °C, and catalyst inks were sprayed onto the GDLs using an airbrush. To achieve a uniform coating on the catalyst layer, the hotplate was rotated during spraying process. Catalyst loadings of 1 mg cm^−2^ and 5 mg cm^−2^ were achieved for Pt-CoP@CNTs/CeO_2_-CoP@NCs (cathode) and CoP@CNTs/CeO_2_-CoP@NCs (anode), respectively. For comparison studies, the AEMWE with commercial catalysts was assembled using Pt/C (20 wt% Pt loading) as the cathode with the catalyst loadings of 1 mg cm^−2^, and RuO_2_ (≥99.9 wt%) as the anode with the catalyst loadings of 5 mg cm^−2^. The activated membranes were sandwiched between the GDLs of the cathode and anode, ensuring that the catalyst-containing layers faced the membrane. Additionally, to prevent damage to the Sustainion membrane, an additional gasket was required on the anode side.

**AEMWE Stack Operation.** LSVs of the activated AEMWE were recorded from 1.0 to 2.5 V at a scan rate of 1 mV^−1^. Durability testing was conducted at 1 A cm^−2^ for 1400 h. During the tests, the AEMWE was supplied in 1.0 M KOH solution at a flow rate of 45 mL min^−1^ and operated at 60 °C under ambient pressure by applying Water Electrolyzer System (DAIHAN Scientific Co., Ltd). iR compensation was not implemented during the AEMWE test.

**Estimate of cell efficiency and the cost of H_2_ per kilogram of hydrogen.** The volume of produced H_2_ from Pt-CoP@CNTs/CeO_2_-CoP@NCs_(-)_||CoP@CNTs/CeO_2_-CoP@NCs_(+)_ in the AEMWE was calculated as following equations:

${Mass}_{H_{2}}=\left( H_{2} production rate \right) \times Electrolyzer area \times t \times(Molar mass H_{2})$ (3)

$V_{H_{2}}= \frac{{Mass}_{H_{2}}}{\rho}$ *(4)*

Where the theoretical H_2_ production rate is 5.18 × 10^-6^ mol·H_2_/(cm^2^·s) at 1 A·cm^−2^; electrolyzer area is 4 cm^2^; t is the operation time (s); molar mass H_2_ is 2.016 g mol^–1^; $\rho$ is 0.09 g L^–1^.

The energy efficiency (*ε*_cell_) and energy consumption of the AEMWE can be obtained as follows:

| $\varepsilon_{cell} \left( \% \right)=\frac{E_{output}}{E_{input}}= \frac{V_{H_{2}} \times H_{c}}{E_{input}}\times100$ | (5) |
| --- | --- |
| $E_{input}=Cell voltage \times Time \times Cunrrent density \times Electrolyzer area$ | (6) |
| $Energy consumption =\frac{AEMWE power \times Electrolyzer area \times t}{{Mass}_{H_{2}}}$ | \| (7) \| \| --- \| |

Where, $V_{H_{2}}$ is the hydrogen gas volume, H_c_ is the calorific value of hydrogen (10.8 × 10^6^ *J* m^−3^, lower heating value), and the AEMWE power $\left( W \mathrm{cm}^{-2} \right)$= cell current density × cell voltage.

Cost of H_2_ (take $0.02/kW h) can be determined from the following equation:

| $Cost (H_{2} {kg}^{-1}) = Energy consumption \times Electricity bill$ | (8) |
| --- | --- |

**Turn-over frequency (TOF) calculation.** The active site number (n) and the TOF per-site (s^-1^) were calculated using the equations:

| $n=\frac{Q}{2F}=\frac{It}{2F}=\frac{IV}{2Fv}$ | (9) |
| --- | --- |
| $TOF=\frac{I}{nmF}$ | (10) |

where n was accessed by CV measurement in PBS solution (pH ~7); *F* is the Faraday constant (96485 C mol^-1^); *v* is the scanning rate (0.05 V s^-1^); I is the current during the LSV tests and the electrodes surface area, respectively; m is the number of electrons required to produce one H_2_ (m value for HER is 2) or O_2_ (m value for OER is 4) molecule from H_2_O.

The n values of Pt-Co_3_O_4_@CNTs/CeCoO_x_@NCs, CoP@CNTs/CeO_2_-CoP@NCs, Pt-Co_3_O_4_@CNTs/CeCoO_x_@NCs, and Pt-CoP@CNTs/CeO_2_-CoP@NCs are revealed to be 5.75106392242898E-07, 2.74352537534332E-07, 3.86871645442566E-07, and 1.64513031248381E-07 mol, respectively (Figure S18).

**Faradaic efficiency (FE) calculation.** The Faradaic efficiency of HER and OER was evaluated by the volume of H_2_ and O_2_ gases, which were separately collected by the assembled electrolyzer at the current density of 100 mA cm^-2^.

The theoretical volume of the produced H_2_ and O_2_ gases were can be calculated as follows:

| $V_{t}=\frac{JTt}{PzF}$ | (11) |
| --- | --- |

where, V_t_ is the theoretical gas volume, J is the current density (mA cm^−2^), R is the ideal gas constant (R = 0.08206 L·atm mol^-1^·K^-1^), T is the operating temperature (298 K), t is the operating time (3600 s), P is the ambient pressure (1 atm), z is the number of electrons involved in the reaction per mole of H_2_ gas (z = 2) or O_2_ gas (z = 4), F is the Faraday constant (96485 C mol^-1^).

The FE was calculated using the ratio of actual gas volume (Vₐ) to theoretical gas volume (Vₜ) according to the following equation:

| $FE \left( \% \right)=\frac{V_{a}}{V_{t}}\times100$ | (12) |
| --- | --- |

**The Electrochemical Surface Area (ECSA) calculation**

The electrochemical surface area (ECSA) can be obtained using the following equation:

| $ECSA=\frac{C_{dl}}{C_{s}}$ | (13) |
| --- | --- |

where, C_dl_ represents measured double-layer capacitance in the non-faradaic region, determined through cyclic voltammetry (CV) tests with scan rates ranging from 5 to 100 mV·s^−1^; C_s_ is the specific capacitance, which varies depending on the type of substrate and electrolyte used. In this study, the value of C_s_ in 1.0 M KOH is 1.375 mF·cm^−2^, as obtained from Figure S21 in the SI.

**Details of the Computational Approach**

The electrocatalyst was systematically investigated using density functional theory (DFT) calculations performed with the Vienna Ab initio Simulation Package (VASP). The projector augmented wave (PAW) method was adopted to describe ion–electron interactions [1-3]. To account for dispersion effects, Grimme’s DFT-D3 correction was employed, while the Perdew–Burke–Ernzerhof (PBE) exchange–correlation functional within the generalized gradient approximation (GGA) framework was used for electronic structure calculations. [4]

To describe the OER under alkaline conditions, we applied the standard four-electron mechanism. The sequence of elementary reactions is as follows:

OH^−^ + ^∗^→ OH^*^ + e^−^  (I)

OH^*^+OH^−^ → O^*^+ H_2_O + e^−^ (II)

O^*^+ OH^−^ → OOH^*^+ e^−^ (III)

OOH^*^ + OH^−^ → O_2_ + ^*^ + H_2_O + e^−^ (IV)

In our OER analysis, we considered the adsorbed intermediates *OH, *O, and *OOH on the catalyst’s active sites. The Gibbs free-energy changes for the four elementary steps are denoted ΔG₁, ΔG₂, ΔG₃, and ΔG₄ (Eqs. I–IV). The theoretical overpotential (η) for each structure was then computed using the following equation:

$\eta_{OER}=\frac{\max\left\{ {\Delta G}_{1}, {\Delta G}_{2}, {\Delta G}_{3}, {\Delta G}_{4} \right\}}{e}-1.23\left( V \right)$ (1)

The term $\max\left\{ {\Delta G}_{1}, {\Delta G}_{2}, {\Delta G}_{3}, {\Delta G}_{4} \right\}$ denotes the largest Gibbs free-energy change among the elementary steps. [5]


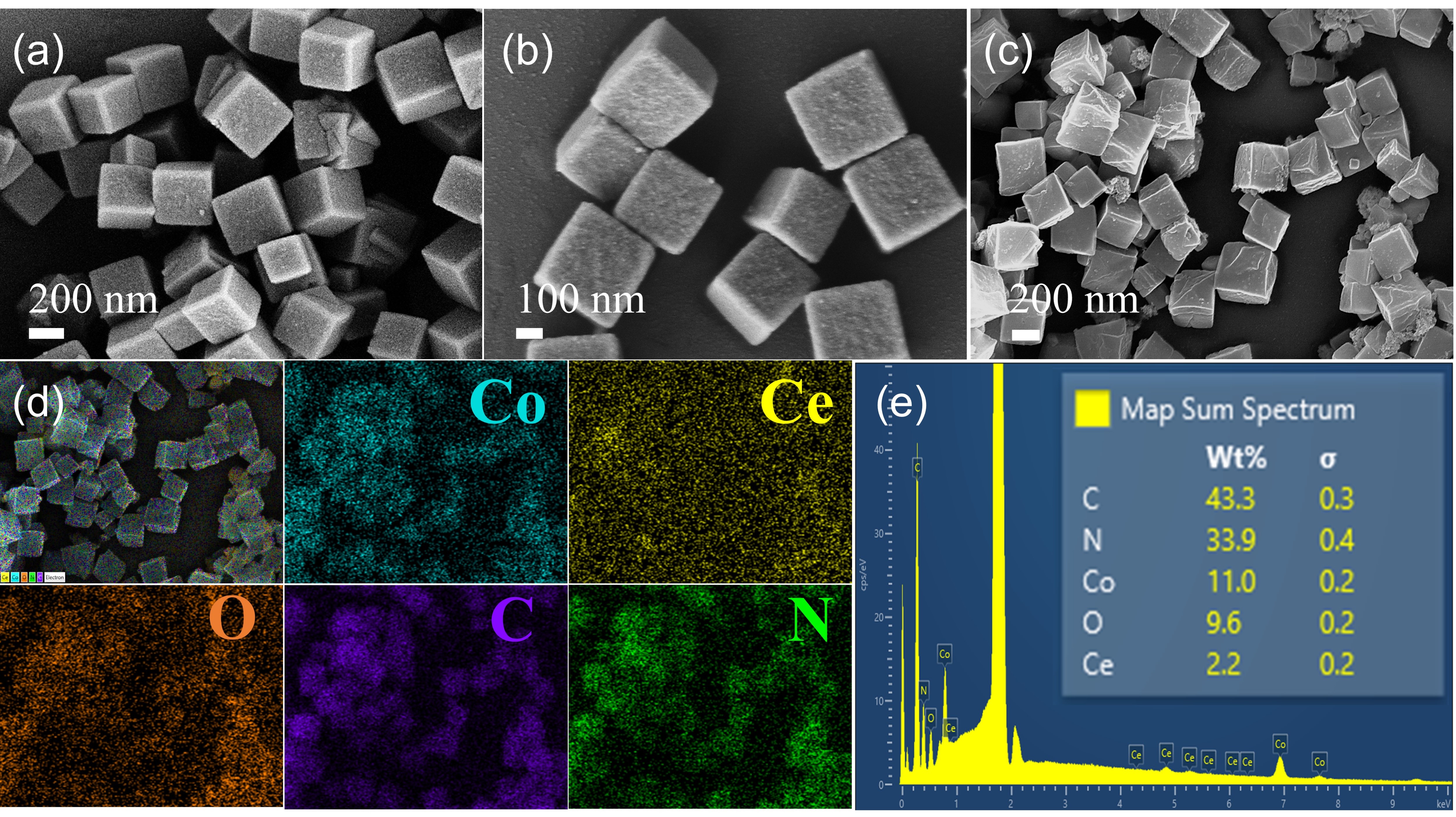


**Figure S1**. (a-b) FE-SEM images at different magnifications, (c-d) SEM-EDS elements mapping images and (e) corresponding EDS spectrum of the CeCo-MOF.

**Figure S2**. XRD pattern of the Co-MoF(ZIF-67) and CeCo-MOF material.


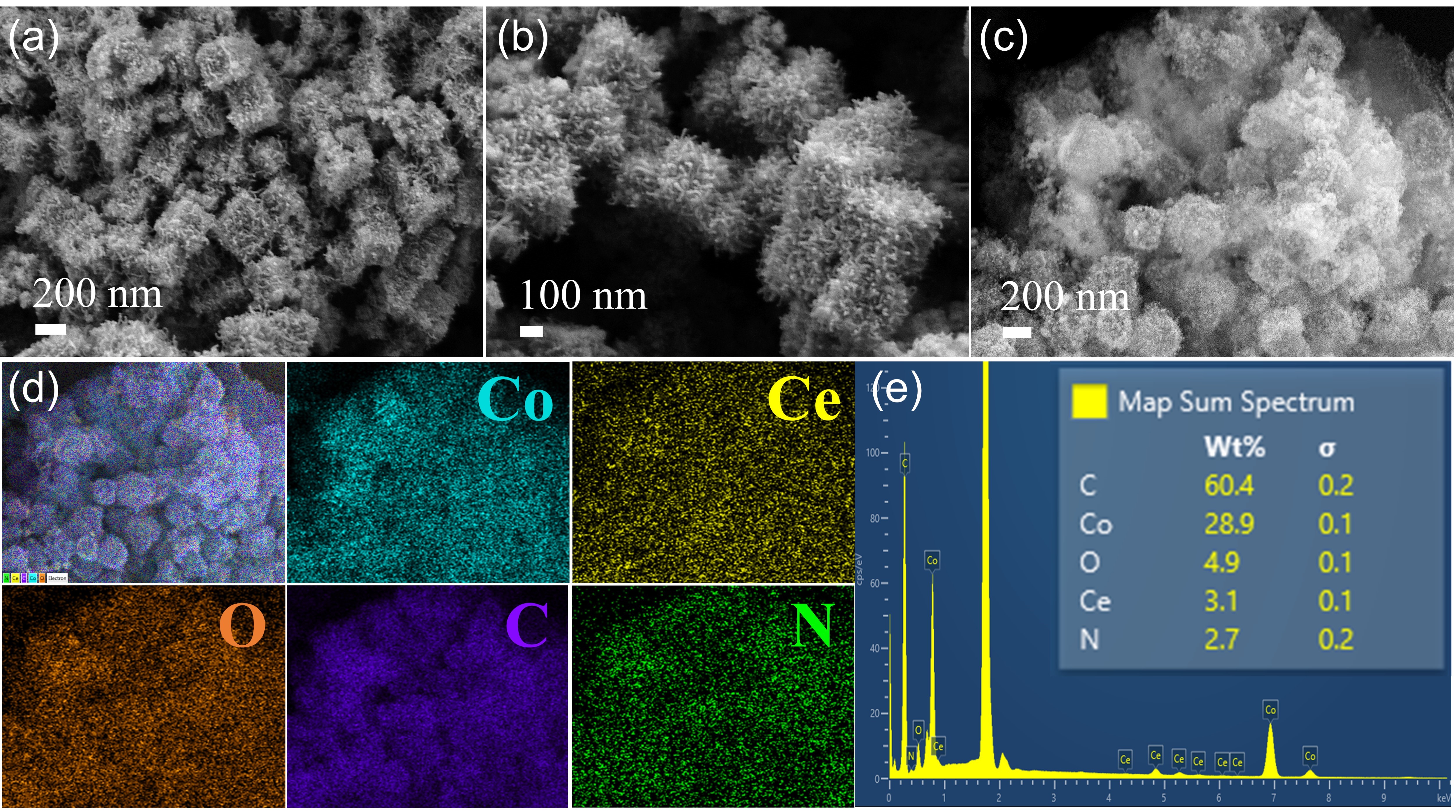


**Figure S3**. (a-b) FE-SEM images at different magnifications, (c-d) SEM-EDS elements mapping images and (e) corresponding EDS spectrum of the Co_3_O_4_@CNTs/CeCoO_x_@NCs.


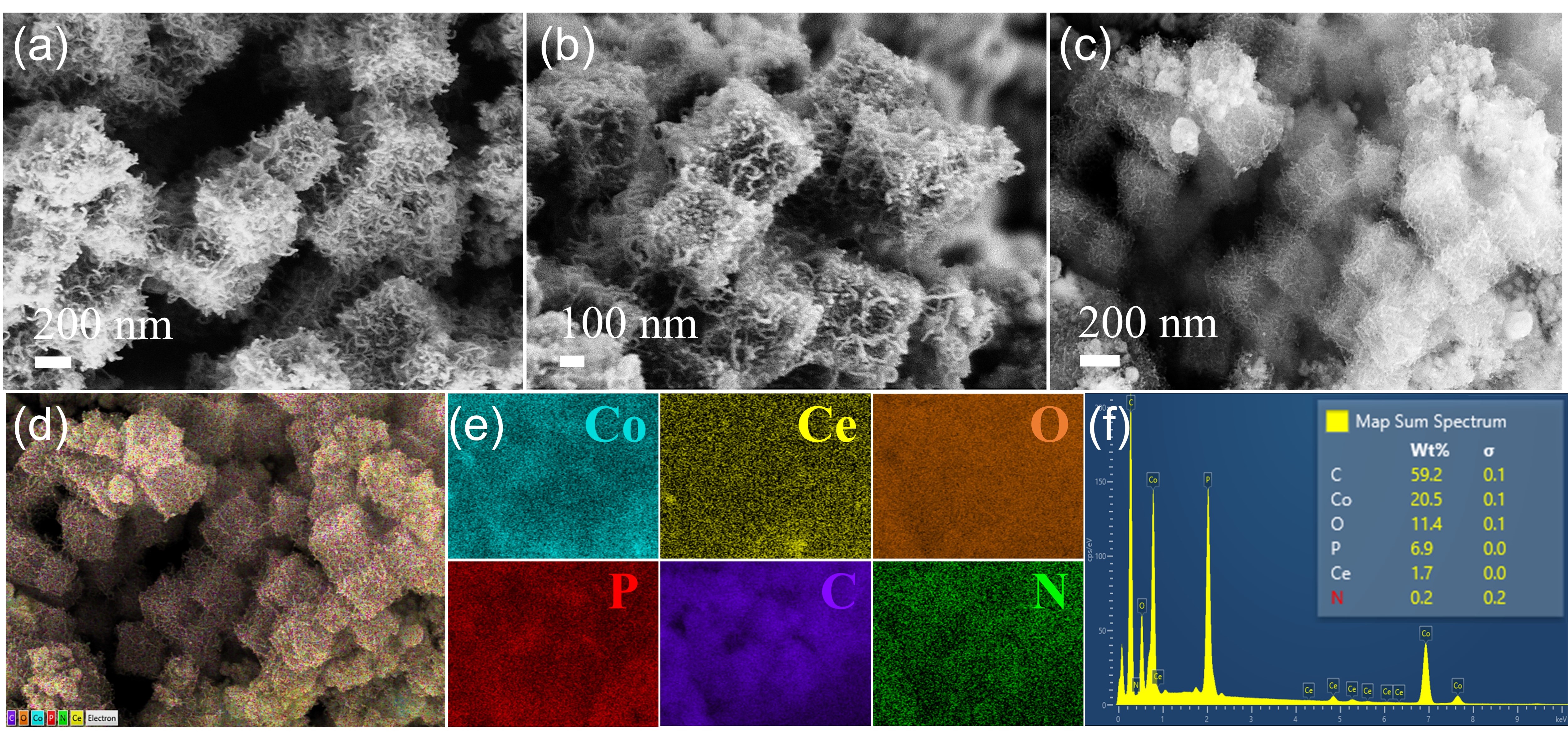


**Figure S4**. (a-b) FE-SEM images at different magnifications, (c-d) SEM-EDS elements mapping images and (e) corresponding EDS spectrum of the CoP@CNTs/CeO_2_-CoP@NCs.


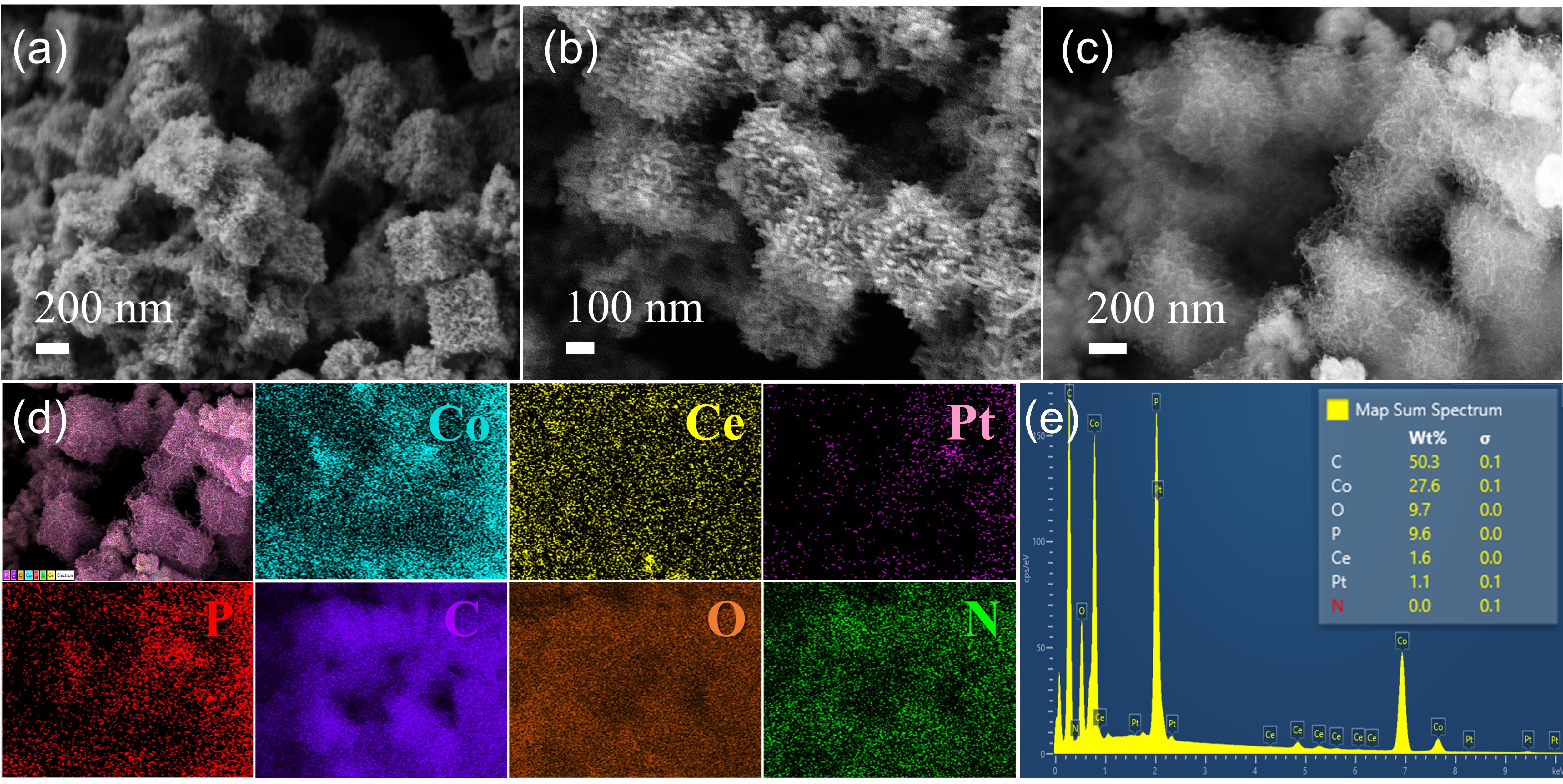


**Figure S5**. (a-b) FE-SEM images at different magnifications, (c-e) SEM-EDS elements mapping images and (f) corresponding EDS spectrum of the Pt-CoP@CNTs/CeO_2_-CoP@NCs-0.1 (0.1 mM).


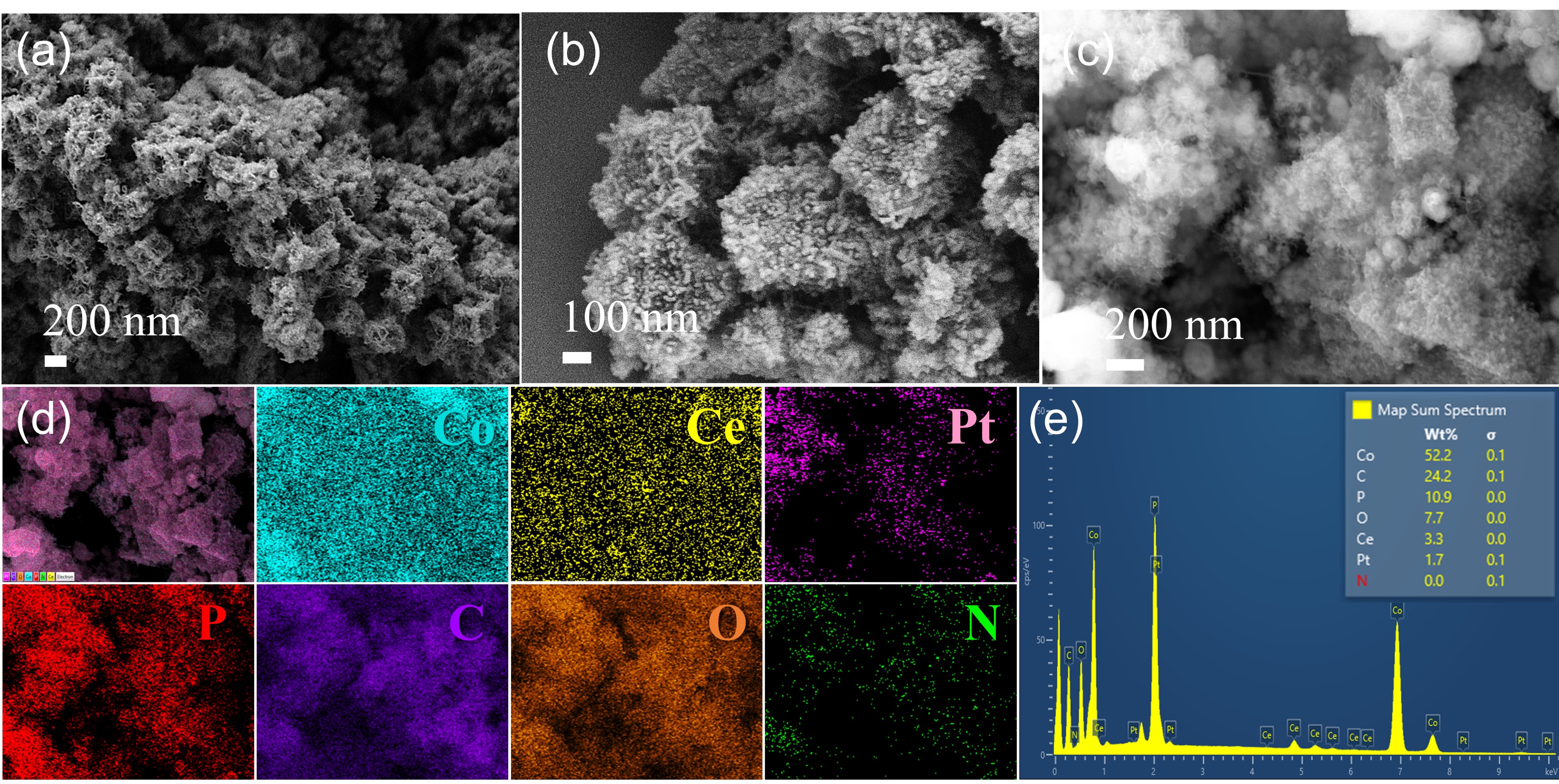


**Figure S6**. (a-b) FE-SEM images at different magnifications, (c-e) SEM-EDS elements mapping images and (f) corresponding EDS spectrum of the Pt-CoP@CNTs/CeO_2_-CoP@NCs-0.4 (0.4 mM).


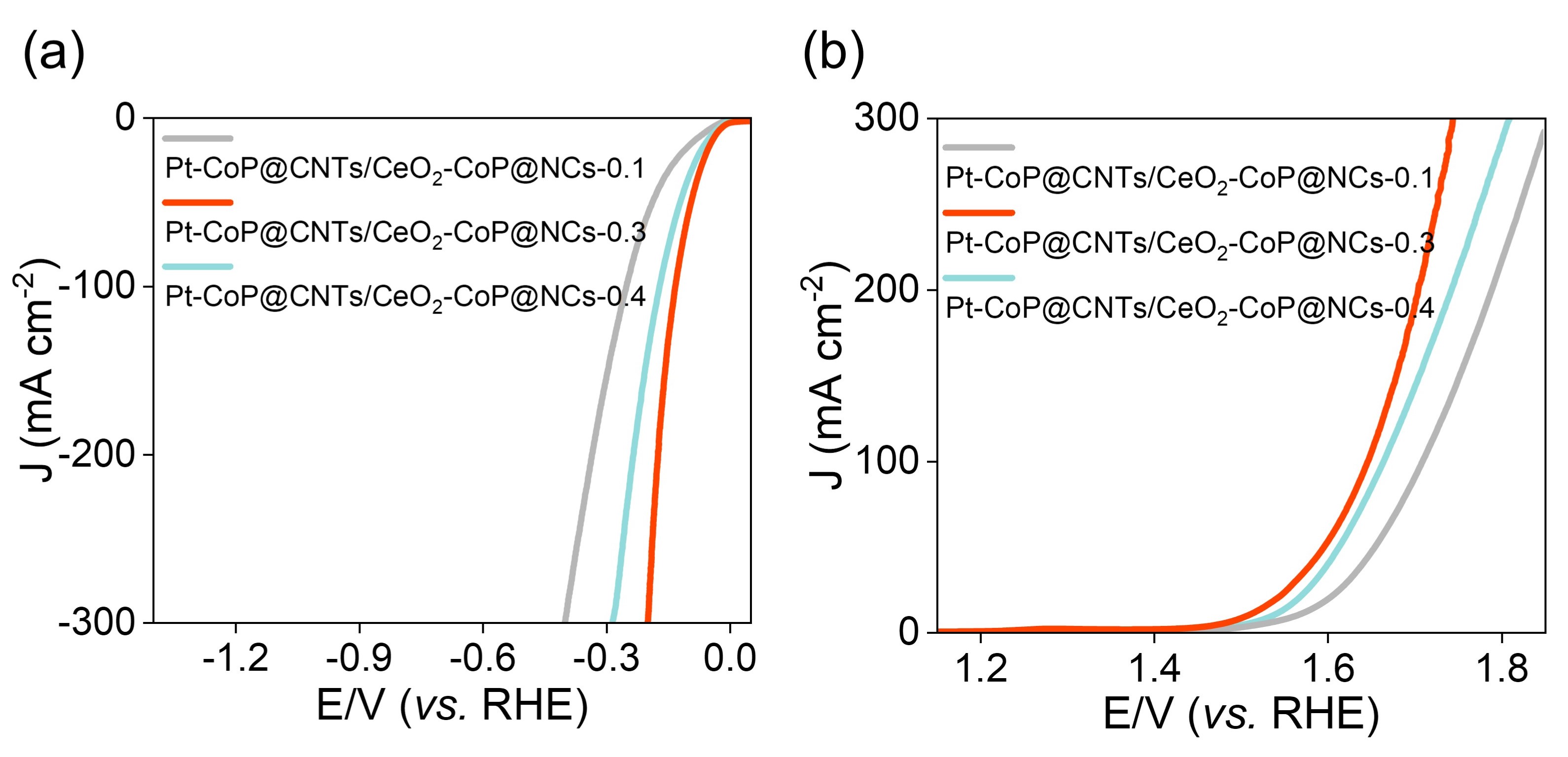


**Figure S7**. (a) HER and (b) OER activities of the Pt-CoP@CNTs/CeO_2_-CoP@NCs achieved by using different amounts of H_2_PtCl_6_ precursor: 0.1, 0.3, and 0.4 mM.


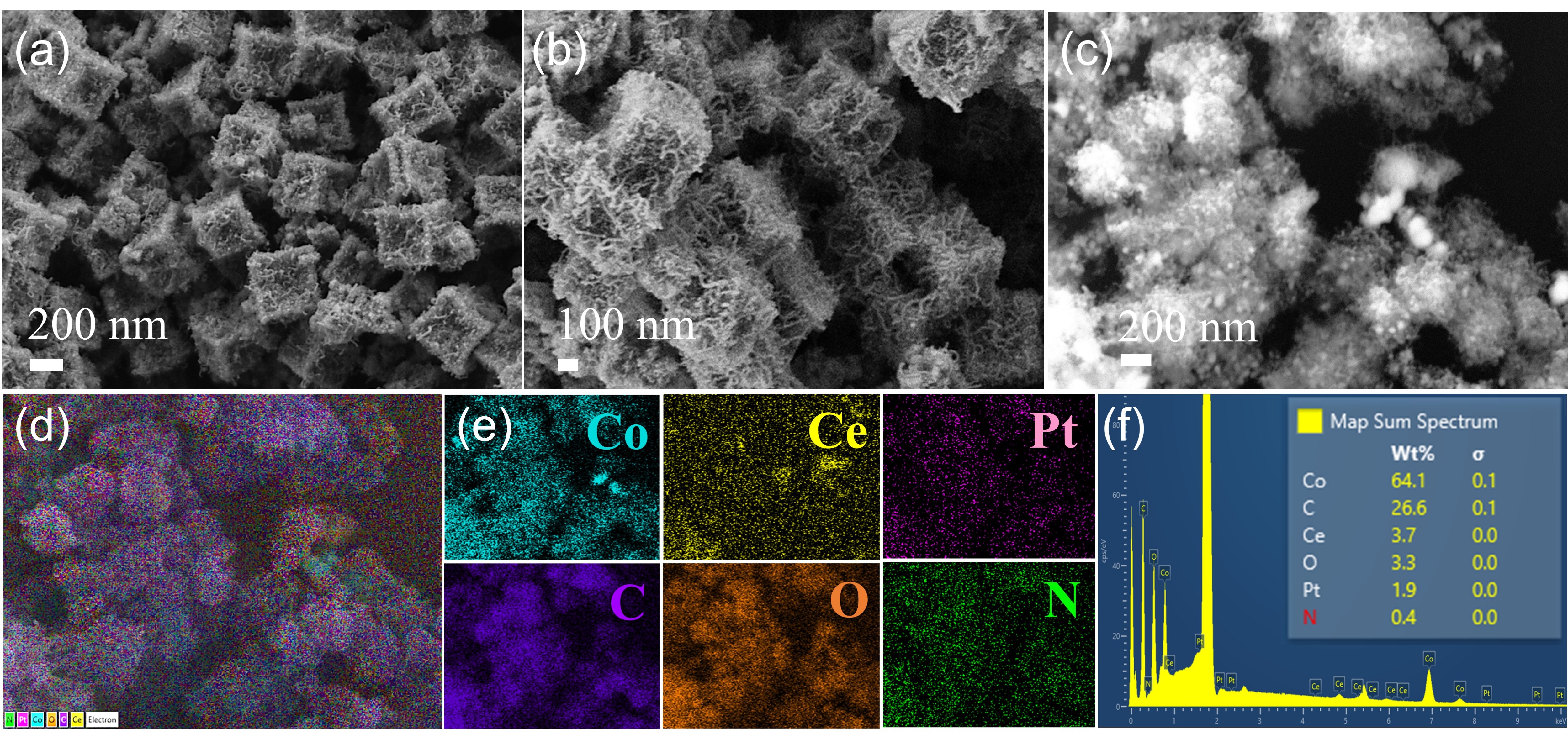


**Figure S8**. (a-b) FE-SEM images at different magnifications, (c-e) SEM-EDS elements mapping images and (f) corresponding EDS spectrum Pt-Co_3_O_4_@CNTs/CeCoO_x_@NCs.

**Figure S9.** OER ΔG diagram for CeO₂/CoP (U = 0 V) at Ce, O, Co, and P adsorption sites with the achievement of their overpotential values.

**Figure S10.** Gibbs free-energy diagram for the OER on Pt-CeO₂/CoP (U = 0 V) at Pt, Ce, O, Co, and P adsorption sites, with corresponding overpotentials.

**Figure S11.** Element-resolved OER overpotential (η) comparison for Pt–CeO₂/CoP vs. CeO₂/CoP at Pt, Ce, O, Co, and P active sites.

**Figure S12**. (a) XPS survey spectra of the Pt-CoP@CNTs/CeO_2_-CoP@NCs, CoP@CNTs/CeO_2_-CoP@NCs, and Pt-Co_3_O_4_@CNTs/CeCoO_x_@NCs materials.

**Figure S13**. Cyclic stability assessment of the Pt-CoP@CNTs/CeO_2_-CoP@NCs catalyst for HER.


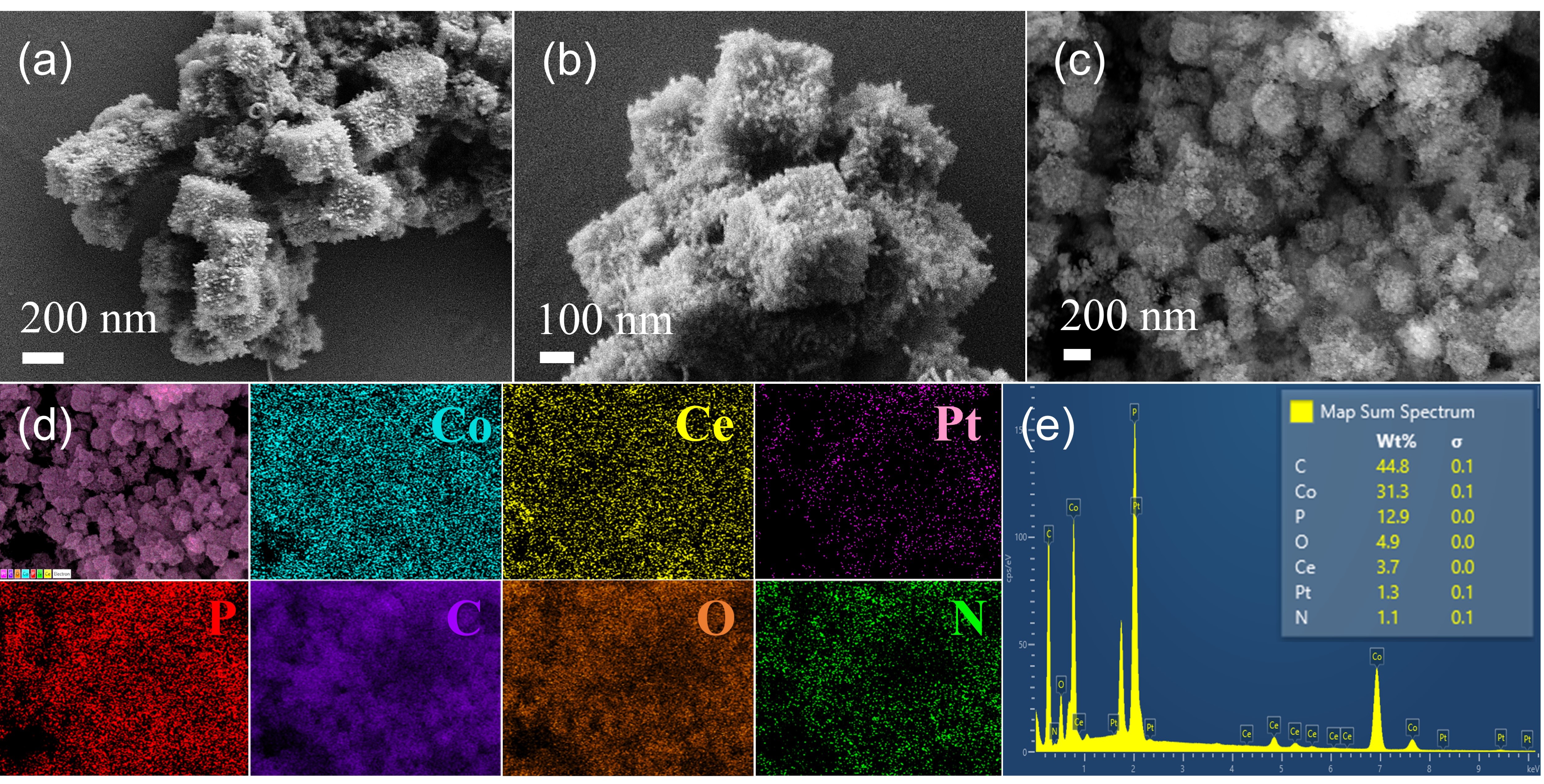


**Figure S14**. (a-b) FE-SEM images at different magnifications, (c-e) SEM-EDS elements mapping images and (f) corresponding EDS spectrum of the post-HER Pt-CoP@CNTs/CeO_2_-CoP@NCs.

**Figure S15.** XRD pattern of the post-HER Pt-CoP@CNTs/CeO_2_-CoP@NCs.


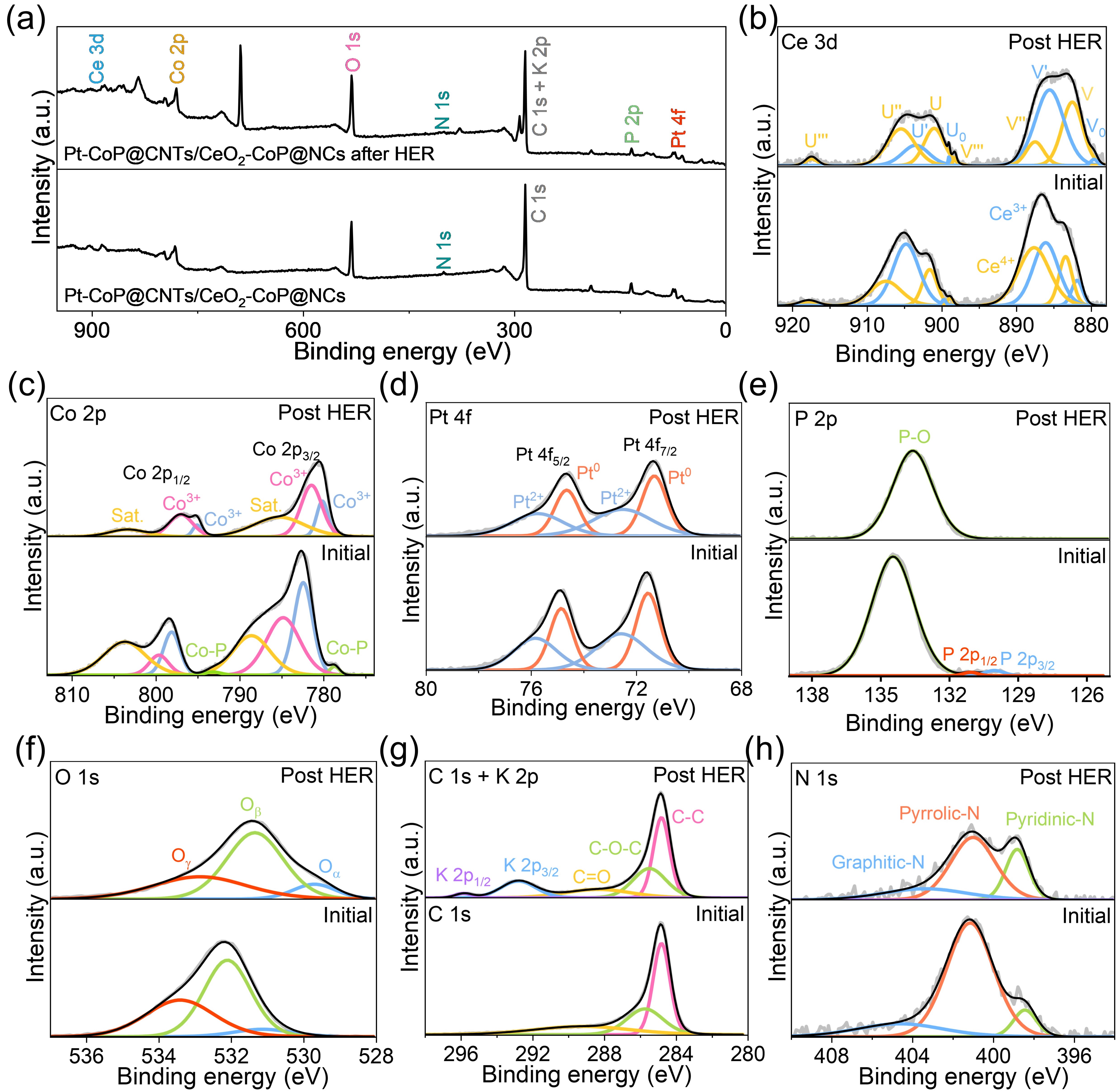


**Figure S16.** (a) Survey XPS spectra and high resolution XPS spectra of (b) Ce 3d, (c) Co 2p, (d) Pt 4f, (e) P 2p, (f) O 1s, (g) C 1s and (h) N 1s binding energies achieved from intial and post-HER Pt-CoP@CNTs/CeO_2_-CoP@NCs.

**Figure S17**. Cyclic stability assessment of the CoP@CNTs/CeO_2_-CoP@NCs catalyst for OER.


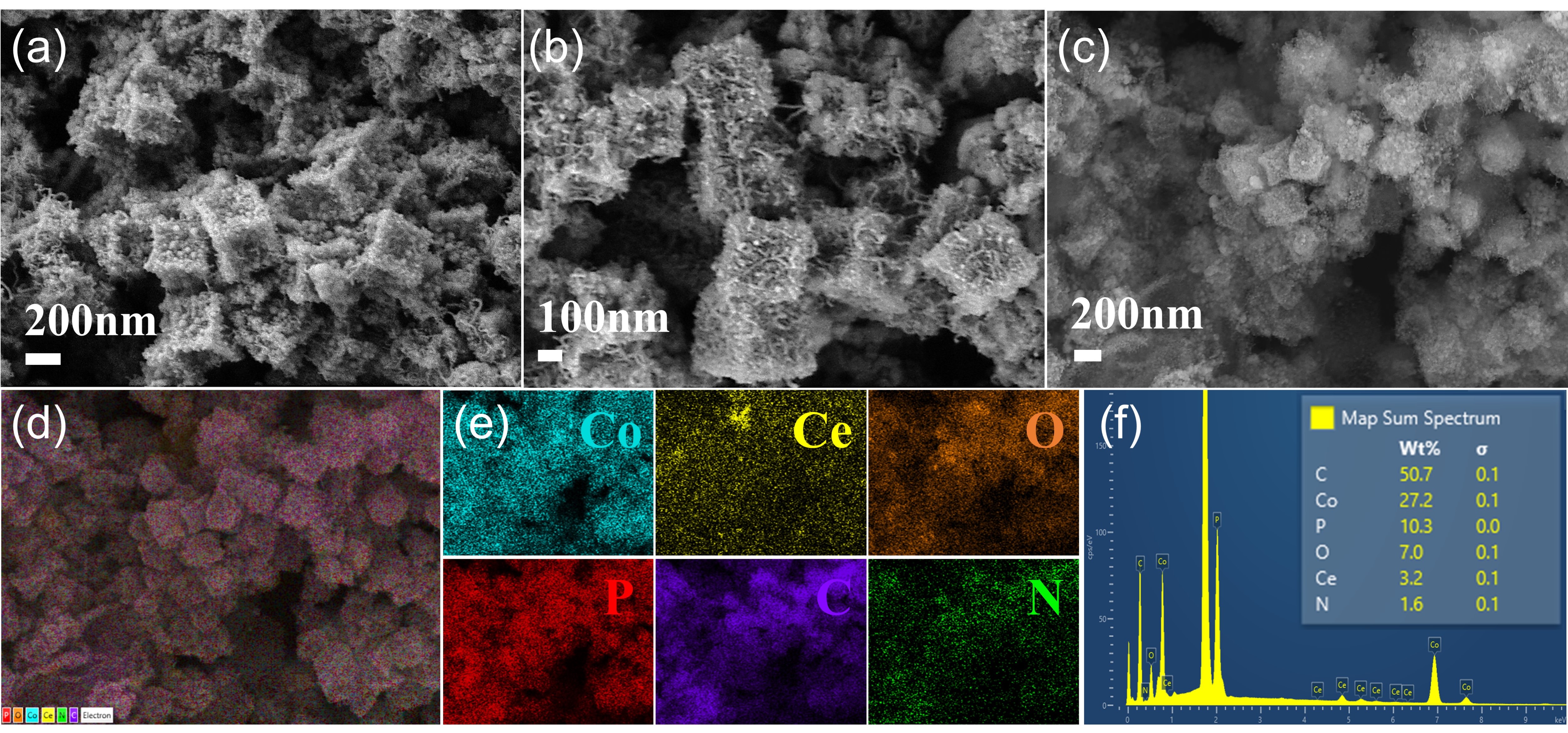


**Figure S18**. (a-b) FE-SEM images at different magnifications, (c-e) SEM-EDS elements mapping images and (f) corresponding EDS spectrum of the post-OER CoP@CNTs/CeO_2_-CoP@NCs.

**Figure S19.** XRD pattern of the post-OER CoP@CNTs/CeO_2_-CoP@NCs.


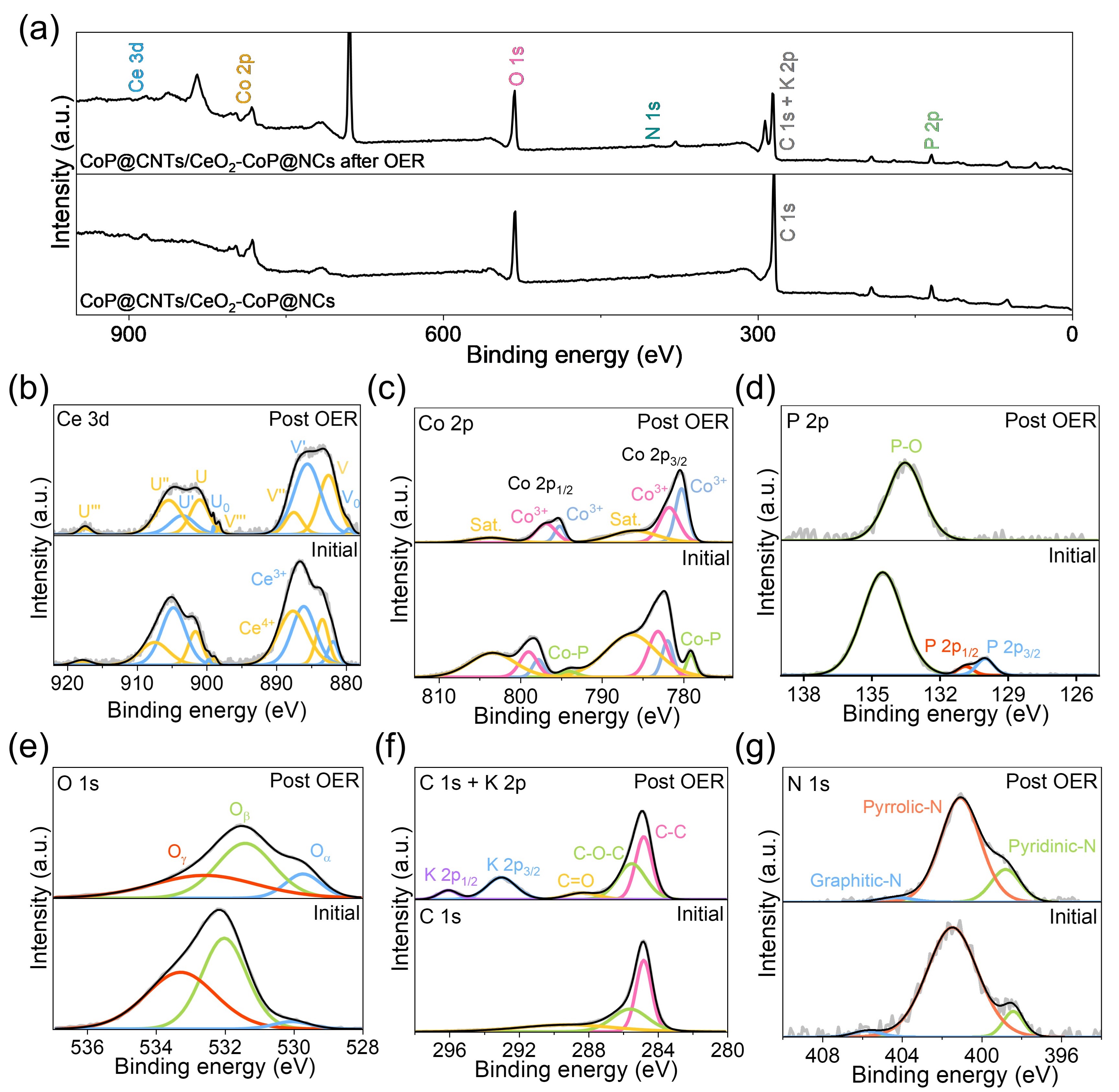


**Figure S20.** (a) Survey XPS spectra and high resolution XPS spectra of (b) Ce 3d, (c) Co 2p, (d) P 2p, (e) O 1s, (f) C 1s and (g) N 1s binding energies achieved from intial and post-OER CoP@CNTs/CeO_2_-CoP@NCs.

**
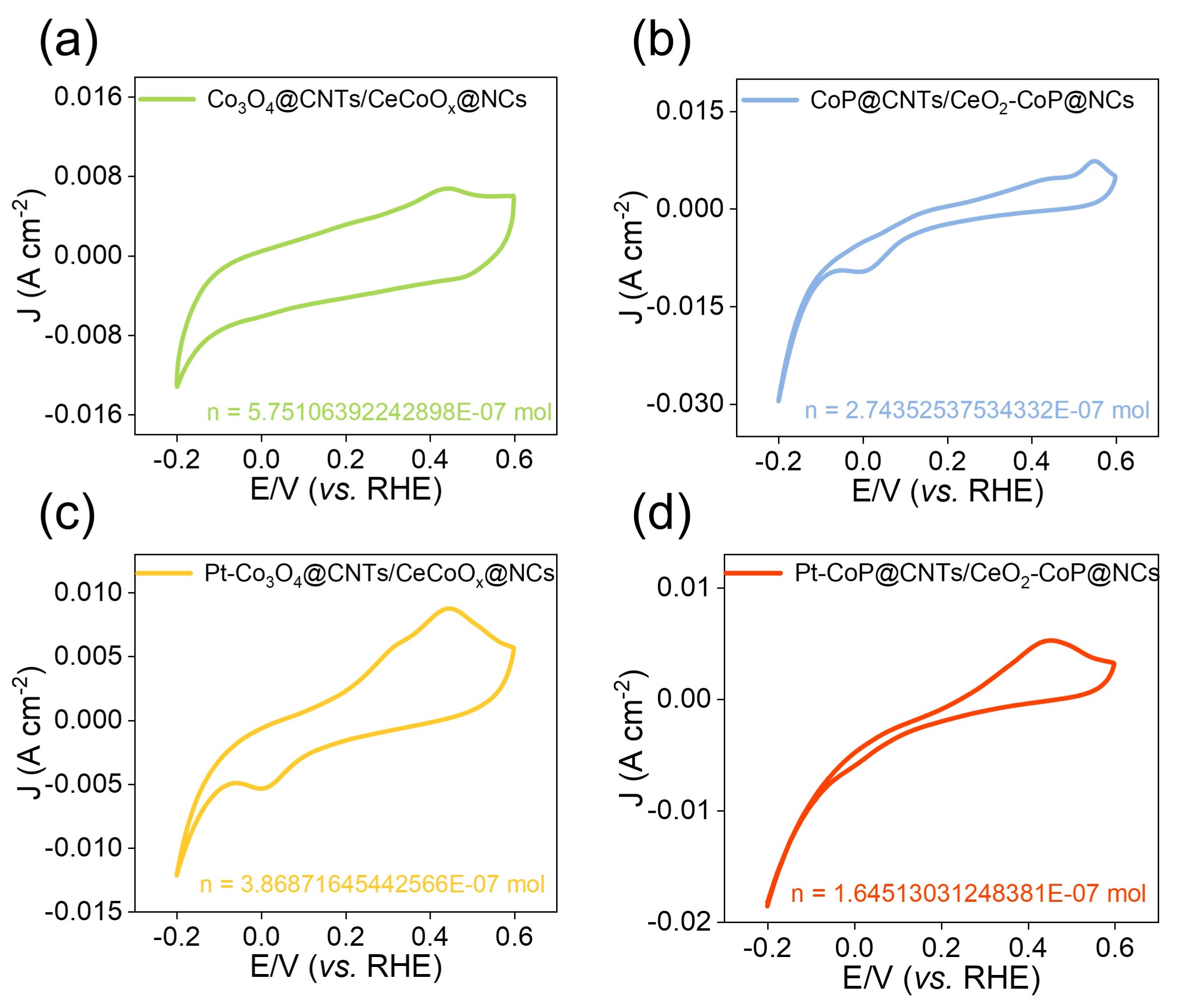
**

**Figure S21.** CV curves of (a) Co_3_O_4_@CNTs/CeCoO_x_@NCs, (b) CoP@CNTs/CeO_2_-CoP@NCs, (c) Pt-Co_3_O_4_@CNTs/CeCoO_x_@NCs, and (d) Pt-CoP@CNTs/CeO_2_-CoP@NCs in PBS solution (pH~7) at a scan rate of 0.05 V s^-1^.

**Figure S22**. EIS of different materials.


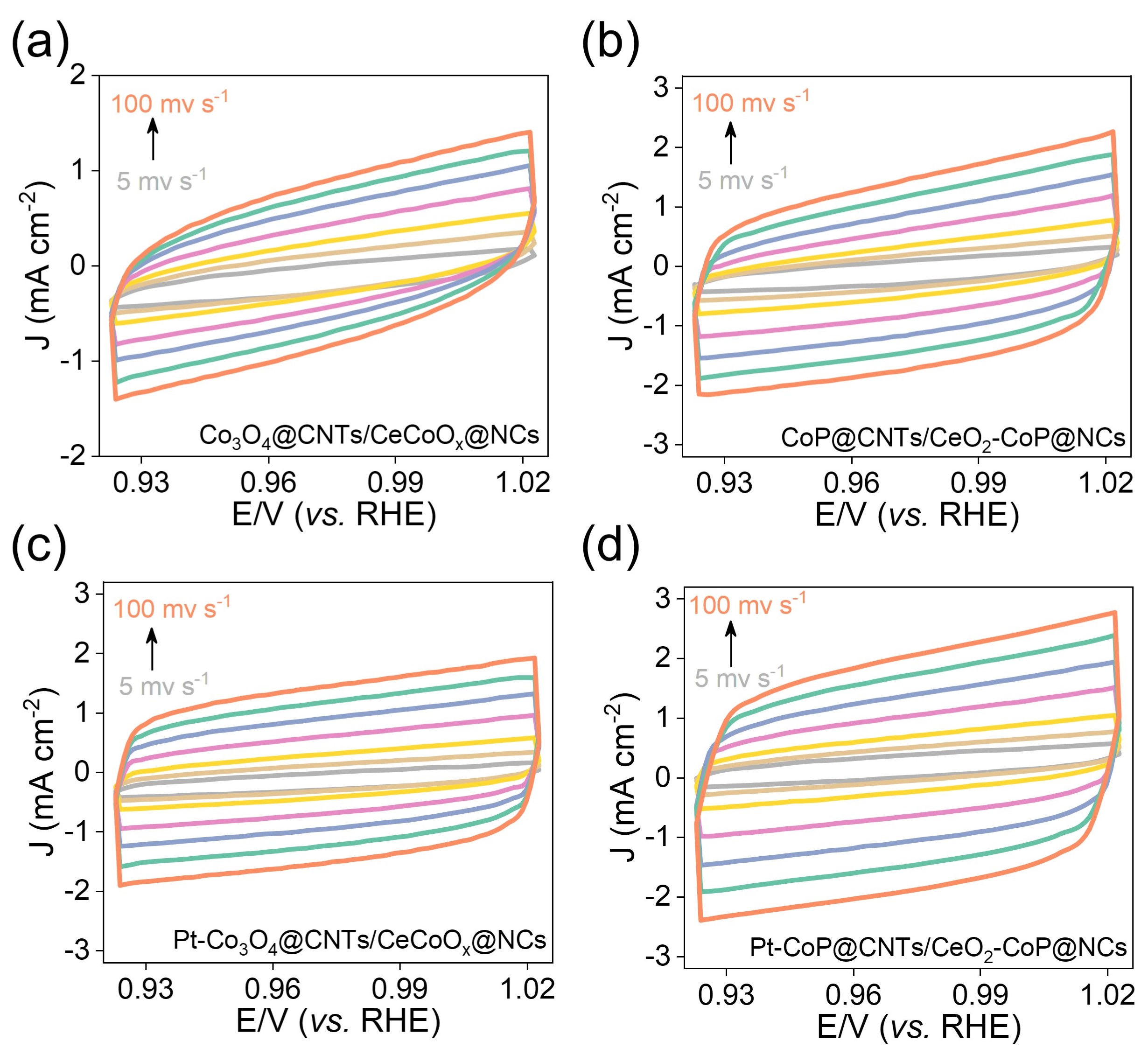


**Figure S23**. CV curves of the materials at different scan rates (5, 10, 20, 40, 60, 80 and 100 mV s^−1^) in a non-Faradaic range.


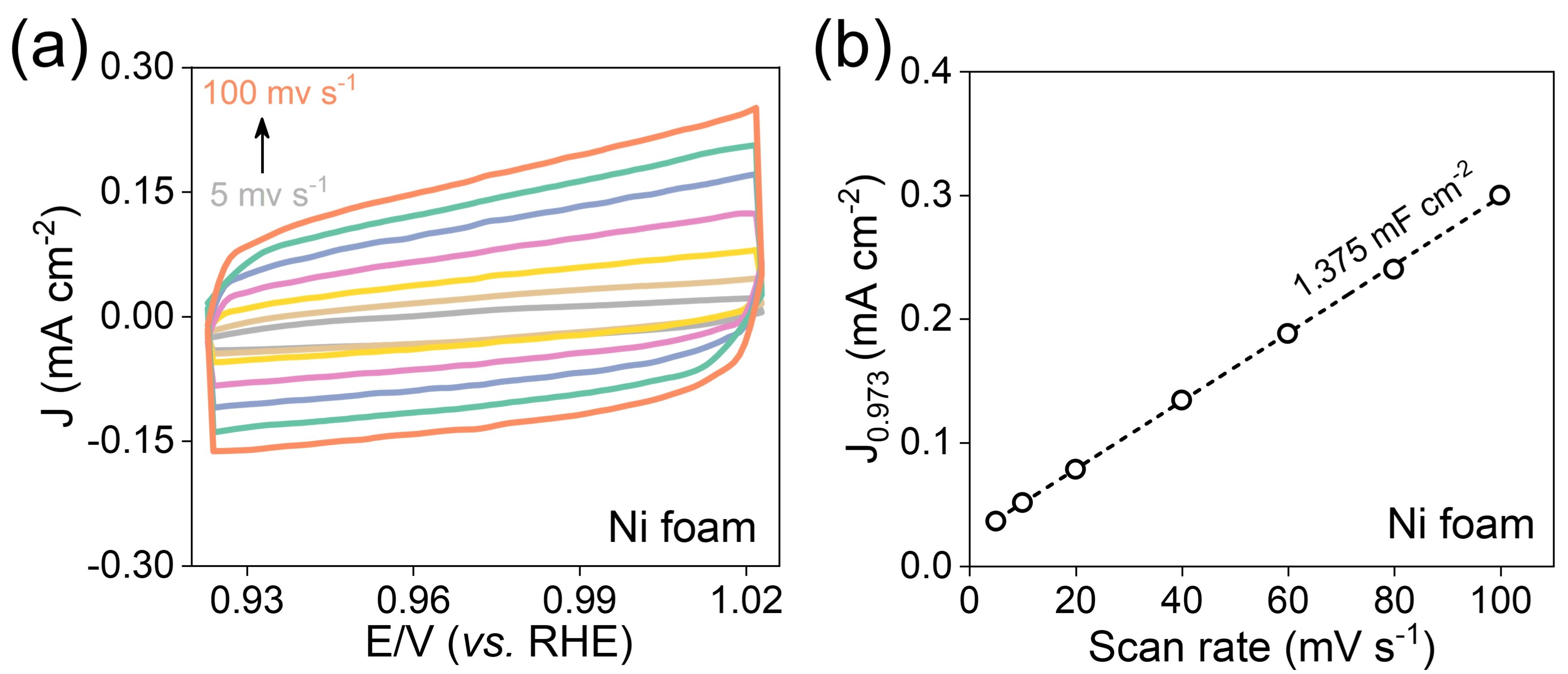


**Figure S24**. (a) CV curves at different scan rates (5, 10, 20, 40, 60, 80 and 100 mV s^−1^) in a non-Faradaic range, and the corresponding (b) C_dl_ plots of Ni foam.


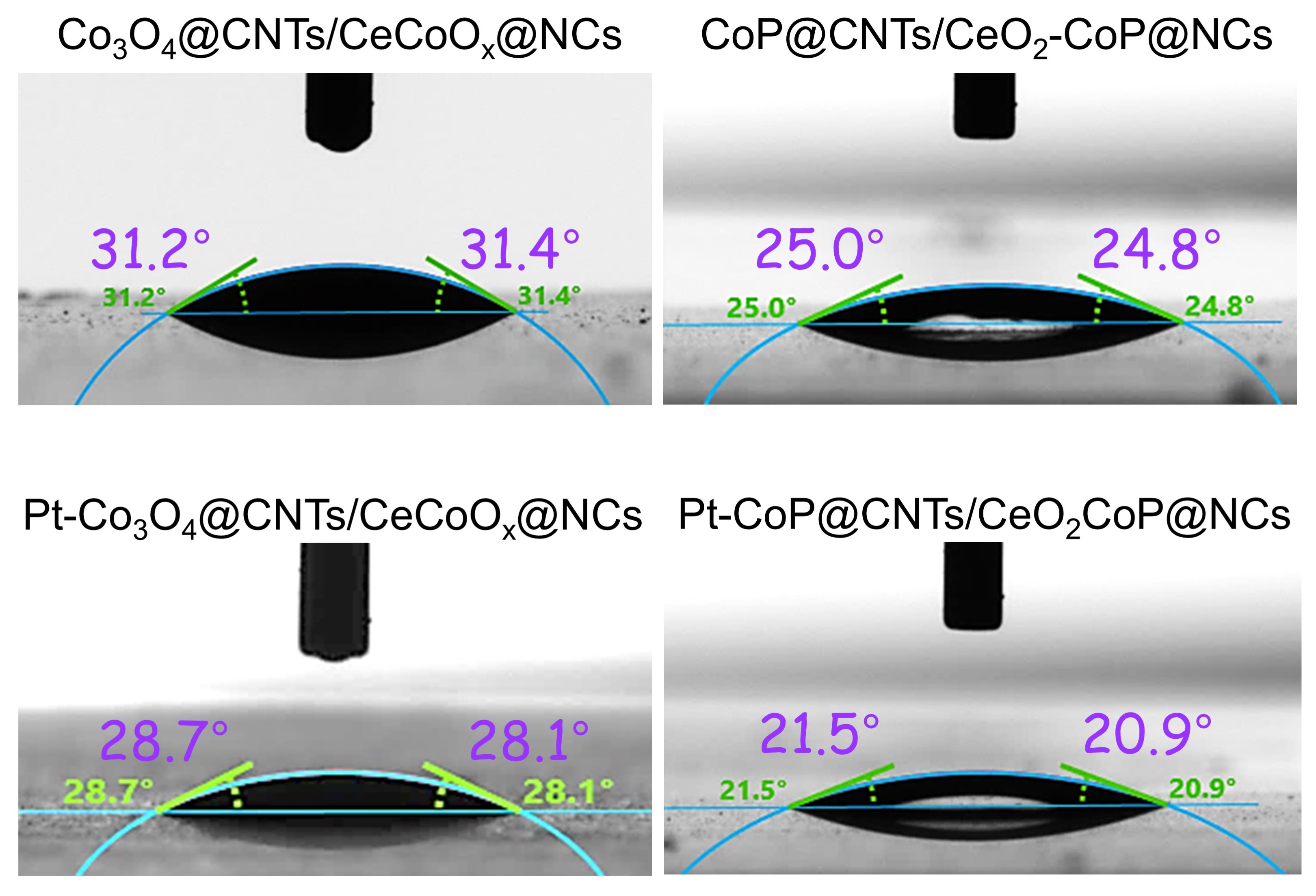


**Figure S25**. WCA measurement of the materials.

**Figure S26**. MA of Pt-CoP@CNTs/CeO_2_-CoP@NCs, Pt-Co_3_O_4_@CNTs/CeCoO_x_@NCs, and Pt/C.

**Figure S27**. MA of Pt-CoP@CNTs/CeO_2_-CoP@NCs_(-)_||CoP@CNTs/CeO_2_-CoP@NCs_(+)_ and Pt/C_(−)_||RuO_2(+)_ cells.

**Table S1.** Comparison of our developed catalyst with previously reported electrocatalysts for HER in 1.0 M KOH electrolyte.

| **Catalyst** | **η_10_**  **(mV)** | **Stability performance** | | | **Reference** |
| --- | --- | --- | --- | --- | --- |
|  |  | **J**  **(mA cm^-2^)** | **Time**  **(h)** | **Retention**  **(%)** |  |
| CoP@Ni_2_P − Fe_2_P | 42 | 10/100 | 40 | 99.5/94.1 | *Appl. Catal. B. Environ. 2023, 338, 123016* |
| Ce-CoP@CC | 81 | 10 | 25 | 92.4* | *Adv. Energy Mater. 2023, 13, 2301162* |
| Co_9_S_8_/Cu_2_S/CF | 165 | 10/50 | 24 | 84.4/84.3 | *ACS Appl. Mater. Interfaces 2021, 13, 9865* |
| NiPt@NVOC | 34.6 | 10/50 | 50 | 94.7/90.3 | *Appl. Catal. B.*  *Environ. 2025, 363, 124801* |
| CoP/CeO_2_-2.5 | 152 | 10 | 16 | 91 | *J. Alloys Compd. 2022, 913, 165334* |
| Ni_2_P@CeO_2_/NF | 62 | 10/100 | 100 | 98.1/94.8 | *Adv. Compos.*  *Hybrid Mater. 2023, 6, 175* |
| NMoNi/SWCNT | 130 | 20 | 20 | 96.4* | *Appl. Catal. B*  *2020, 269,118823* |
| Pt_SA_-PtCo NCs/  N-CNTs-900 | 47 | 10 | 60 | 93.0* | *Small*  *2023, 19, 2304294* |
| Co,Nb-MoS_2_/TiO_2_ HSs | 59 | 10/50 | 30 | 97.3/85.2 | *Nano Energy*  *2021, 82, 105750* |
| CoP@PNC-DoS | 173 | 10 | 30 | 83.0 | *Energy Storage Mater. 2020, 28, 27-36* |
| CeO_2_-NiCoP_x_/NCF | 39 | 100 | 100 | 93.6 | *Appl. Catal. B. Environ. 2022, 316, 121678* |
| P-CoMo_2_S_4_/Co_4_S_3_-Co_2_P | 54 | 10/100 | 40 | 96.8/94.9 | *Appl. Catal. B*  *2024, 344, 123649* |
| NF/Co_4_N@CeO_2_ | 49 | 10 | 100 | 78.0* | *Appl. Catal. B. Environ. 2023, 325, 122364* |
| Co_4_N–CeO_2_/NF-2 | 52 | 100 | 24 | 96.0 | *J. Mater. Chem. A. 2021, 9, 1655-1662* |
| Fe_2_P–CoP/CeO_2_-20 | 43 | 10 | 30 | 94.4* | *Chem. Eng. J.*  *2023, 451, 138550* |
| V-CoP@a-CeO_2_ | 68 | 20 | 60 | 92.6* | *Adv. Funct. Mater.*  *2020, 30, 1909618* |
| NiS/Ni_3_S_2_/CeO_2_ | 85 | 10 | 20 | 88 | *Mater. Today Chem. 2023, 34, 101791* |
| Ce-NiS_2_@NF-1 | 100 | 10 | 24 | 98 | *ACS Appl. Nano Mater. 2024, 7, 8, 9730–9744* |
| Ni_2_P@CeO_2_/NF | 62 | 10/100 | 100 | 98.1/94.8 | *Adv. Compos. Hybrid Mater. 2023, 6, 175* |
| **Pt-CeO_2_/CoP**  **@NC-CNT** | **30** | **10/100** | **70** | **96.0/95.3** | ***This work*** |

*The value is estimated from the original curves shown in the literature.

**Table S2.** Comparison of our developed catalyst with previously reported electrocatalysts for OER in 1.0 M KOH electrolyte.

| **Catalyst** | **η @ J**  **(mA cm^-2^)** | **Stability performance** | | | **Reference** |
| --- | --- | --- | --- | --- | --- |
|  |  | **J**  **(mA cm^-2^)** | **Time**  **(h)** | **Retention**  **(%)** |  |
| 1D-Cu@Co-CoO/Rh | 380 mV@50 | 10 | 10 | 90 | *Small*  *2021, 17, 2103826* |
| Co@Ni_m_P_n_–Ru | 320 mV@50 | 50 | 32 | 98.2 | *Small*  *2024, 20, 2309122* |
| CoTe_2_/CoP | 320 mV@50  354 mV@100 | 20 | 30 | 96.5 | *Appl. Catal. B.*  *Environ. 2023, 329, 122551* |
| CoN_3_ NLF | 390 mV@50*  454 mV@100* | - | 30 | 88.7 | *Energy Storage Mater. 2024, 65, 103184* |
| Co_2_P/CoP@Co@NCNT | 310 mV@50  360 mV@100 | 10 | 50 | 84.9 | *Chem. Eng. J.*  *2022, 430, 132877* |
| Mo-CoOOH | 345 mV@50  360 mV@100 | 45 | 20 | 91 | *Nano Energy*  *2018, 48, 73-80* |
| CoTe_2_@NCFs | 387 mV@50 | 10 | ~6.7 | 93.1 | *Energy Storage Mater. 2024, 71, 103654* |
| Se–NiMoO_4_·xH_2_O | 336 mV@50*  362 mV@100* | 50 | 50 | 96.02 | *Chem. Eng. J.*  *2024, 498, 155287* |
| Co_9_S_8_@Co/Mn-S,N-PC | 342 mV@50* | 10 | 12 | 85.8* | *J. Colloid. Interface Sci. 2022, 608, 2100-2110* |
| Co-N-CCNFMs | 380 mV@50* | 20 | 17 | 98.5 | *Energy Storage Mater. 2022, 47, 365-375* |
| FeCo-MI@TAP-900 | 480 mV@50* | 10 | 15 | 91 | *Energy Storage Mater. 2024, 65, 103106* |
| Co_8_FeS_8_–CoS@CNT | 325 mV@50* | 20 | 36 | 64.4* | *J. Power*  *Sources 2020, 449, 227561* |
| CoO@S-CoTe | 343 mV@50*  362 mV@100* | 100 | 12.5 | 99.4* | *Adv. Sci. 2023, 10, 2206204* |
| FeNi/Co_4_N-NCS | 513 mV@50* | 10 | 50 | 86 | *Energy Storage Mater. 2023, 59, 102783* |
| (NiFeCoMn)_3_S_4_ | 365 mV@50*  423 mV@100* | 10 | 48 | 89.2* | *Adv. Funct. Mater. 2023, 33, 2208170* |
| CeO_2_@CoS/MoS_2_ | 330 mV@50* | 10 | 12 | 82.0* | *Chem. Eng. J.*  *2021, 420, 127595* |
| Co@0.3 g FeCoTe_2_-200 | 322@50 | 10 | 15 | 98.2* | *Dalton Trans. 2023,52, 7906-7916* |
| Co/CoP@HOMC | 409 mV@50*  480 mV@100* | 10 | 20 | 96.2* | *Adv. Energy Mater. 2021, 11, 2102134* |
| FeCo-NPC | 375 mV@50*  419 mV@100* | 10 | 20 | 84.7 | *Energy Storage Mater. 2023, 59, 102772* |
| **CoP@CNTs/CeO_2_-CoP@NCs** | **320 mV@50**  **360 mV@100** | **50/100** | **70/70** | **95.4/94.7** | ***This work*** |

*The value is estimated from the original curves shown in the literature.

**Table S3.** Comparison of previously reported electrolyzer cells for the cell voltage (@10 mA cm^−2^) and stability during long-term water splitting.

| **Catalyst** | **Cell**  **Voltage**  **(V)** | **Electrolyte** | **Stability performance** | | | **Reference** |
| --- | --- | --- | --- | --- | --- | --- |
|  |  |  | **J**  **(mA cm^-2^)** | **Time**  **(h)** | **Retention**  **(%)** |  |
| V-Ce/CoFe LDH | 1.65 | 1.0 M KOH | 10 | 60 | 47.2* | *J. Mater. Chem. A. 2020, 8, 2490-2497* |
| CoP@FeCoP/NC YSMPs | 1.68 | 1.0 M KOH | 10 | 20 | 96.0* | *Chem. Eng. J.*  *2021, 403, 126312* |
| Pt_SA_-Mn,Fe-Ni LDHs | 1.58 | 1.0 M KOH | 50 | 50 | 96.0 | *ACS Nano*  *2024, 18, 16222-16235* |
| CC@Co(OH)_2_@CoSe | 1.71 | 1.0 M KOH | 10 | 50 | 96.2 | *Nanoscale Adv.*  *2020, 2, 792-797* |
| Al,Fe-CoP/RGO | 1.66 | 1.0 M KOH | 10 | 10 | 98.5* | *Chem. Eng. J.*  *2021, 421,127856* |
| 1D-Cu@Co-CoO/Rh | 1.62 | 1.0 M KOH | 10 | 10 | 73.0* | *Small*  *2021, 17, 2103826* |
| Co_9_S_8_/Cu_2_S/CF | 1.60 | 1.0 M KOH | 10 | 8 | 92.0 | *ACS Appl. Mater. Interfaces 2021, 13, 9865* |
| CN@CNPt_(-)_ **\|\|** CN@CNP_(+)_ | 1.53 | 1.0 M KOH | 100 | 30 | 99.0 | *Nano Energy*  *2024, 123, 109413* |
| RuP/CoNiP_4_O_12_/CC | 1.56 | 1.0 M KOH | 10 | 200 | 90.4* | *Appl. Catal. B. Environ. 2023, 328, 122447* |
| Pt_SA_–NiMoO_4_·xH_2_O_(–)_ **\|\|** Se–NiMoO_4_·xH_2_O_(+)_ | 1.598 | 1.0 M KOH | 50 | 50 | 92.0 | *Chem. Eng. J.*  *2024, 498, 155287* |
| Ni_2_P@CeO_2_/NF | 1.55 | 1.0 M KOH | 100 | 100 | 94.2 | *Adv. Compos. Hybrid Mater. 2023, 6, 175* |
| Ce_0.1_Ni_0.85_Se | 1.73 | 1.0 M KOH | 20 | 24 | 74.0* | *ACS Appl. Nano Mater. 2024, 7, 8, 9730–9744* |
| Co,Nb-MoS_2_/TiO_2_ HSs | 1.59 | 1.0 M KOH | 10 | 60 | 89.2 | *Nano Energy*  *2021, 82, 105750* |
| CoP/EEBP | 1.67 | 1.0 M KOH | 10 | 10 | 87.8* | *Chem. Eng. J.*  *2020, 395, 124976* |
| Ce-CoP@CC | 1.57 | 1.0 M KOH | 10 | 27 | 79.0* | *Adv. Energy Mater. 2023, 13, 2301162* |
| CoP@PNC-DoS | 1.74 | 1.0 M KOH | 10 | 30 | 90.0 | *Energy Storage Mater. 2020, 28, 27-36* |
| Co_2_P/CoP@Co@NCNT | 1.60 | 1.0 M KOH | 10 | 12 | 95.1* | *Inorg. Chem. 2023, 62, 44, 18189–18197* |
| Co@CoP@NPCNTs | 1.63 | 1.0 M KOH | 20 | 18* | 95.5* | *Small*  *2020, 16, 2002114* |
| Cr-CoP/CP | 1.59 | 1.0 M KOH | 10 | 30 | 85.0 | *Chem. Eng. J.*  *2021, 425, 130651* |
| CoP/Co(OH)_2_@NF | 1.58 | 1.0 M KOH | 50 | 12 | 97.9* | *Inorg. Chem. 2023, 62, 44, 18189–18197* |
| V-CoP@a-CeO_2_ | 1.56 | 1.0 M KOH | 20 | 35 | 93.2* | *Adv. Funct. Mater. 2020, 30, 1909618* |
| Co3Fe/Ce0.025 | 1.70 | 1.0 M KOH | 10 | ＞24 | 93.3* | *Inorg. Chem. 2023, 62, 21, 8347–8356* |
| Co_x_P@NC | 1.71 | 1.0 M KOH | 10/50 | 10 | 85.3/85.6* | *Carbon*  *2019, 145, 694-700* |
| CoP-InNC@CNT | 1.58 | 1.0 M KOH | 11* | 15 | 87.8 | *Adv. Sci.*  *2020, 7, 5 1903195* |
| CoFeP@C/NF-2 | 1.59 | 1.0 M KOH | 10 | 20 | 82.0 | *Colloid Surf. A. 671 2023, 131688* |
| Ni–Co–P HNBs | 1.62 | 1.0 M KOH | 10 | 20 | 93.4 | *Energy Environ. Sci. 2018, 11, 872-880* |
| hcp-Co@NC | 1.58 | 1.0 M KOH | 10 | 36 | 93.9* | *Appl. Catal. B*  *2020, 266, 118621* |
| O-CoP-2 | 1.60 | 1.0 M KOH | 15* | 18 | 95.3* | *Adv. Funct. Mater. 2020, 30, 1905252* |
| P − CoMo_2_S_4_/Co_4_S_3_–Co_2_P | 1.55 | 1.0 M KOH | 50 | 45 | 95.6 | *Appl. Catal. B*  *2024, 344, 123649* |
| CoFeN_x_HNAs/NF | 1.59 | 1.0 M KOH | 10 | 50 | 82.1* | *ACS Appl. Mater. Interfaces 2020, 12, 26, 29253–29263* |
| Co@Ni_m_P_n_–Ru | 1.57 | 1.0 M KOH | 50 | 50 | 90 | *Small*  *2024, 20, 2309122* |
| **Pt-CoP@CNTs/CeO_2_-CoP@NCs_(-)_ \|\| CoP@CNTs/CeO_2_-CoP@NCs_(+)_** | **1.52** | **30% KOH** | **100** | **150** | **97.7** | ***This work*** |
| **Pt-CoP@CNTs/CeO_2_-CoP@NCs_(-)_ \|\| CoP@CNTs/CeO_2_-CoP@NCs_(+)_** | **1.53** | **1.0 M KOH** | **100** | **150** | **96.1** | ***This work*** |

* The value is estimated from the original curves shown in the literature.

**Table S4.** Comparison of our developed AEMWE with state-of-the-art AEMWEs reported in current research.

| **Cathode \|\| Anode** | **Electrolyte,**  **temperature** | **AEMWEs performance** | | **Stability performance** | | **Reference** |
| --- | --- | --- | --- | --- | --- | --- |
|  |  | **J**  **(A cm^-2^)** | **Cell voltage**  **(V)** | **J**  **(A cm^-2^)** | **Time**  **(h)** |  |
| Pt/C \|\| Ni_2_P/Ni_7_S_6_ | 1.0 M KOH  at 75 °C | 1 | 1.88 | 1 | 140 | *Appl. Catal. B*  *2023, 330, 122633* |
| Pt/C \|\| S-CoCuCO_x_/NF | 1.0 M KOH  at 60°C | 1 | 1.87 | 1 | 110 | *ACS Appl. Mater. Interfaces 2023, 15, 39, 45756–45763* |
| Pt@S-NiFe LDH \|\| S-NiFe LDH | 1.0 M KOH  at 25 °C | 0.5 | 2.5 | 0.5 | 200 | *Adv. Mater. 2023,*  *35, 2208209* |
| Pt-AC/Cr-N-C \|\| NiFe LDH | 1.0 M KOH  at 80 °C | 1 | 1.9 | 0.5 | 100 | *J. Am. Chem. Soc. 2023, 145, 39, 21432–21441* |
| Pt_SA_-Mn,Fe-Ni LDHs \|\| Pt_SA_-Mn,Fe-Ni LDHs | 1.0 M KOH  at 60 °C | 0.5/1 | 1.79/1.97 | 0.5 | 600 | *ACS Nano 2024, 18, 16222-16235* |
| NiFeCoP_x_ \|\| NiFeCo LDH | 1.0 M KOH  at 50 °C | 0.5/1 | 1.72/1.84 | 0.5 | 100 | *Appl. Catal. B 2021, 294, 120246* |
| NFM-OV_R_/NF \|\| NFM-OV_R_/NF | 30% KOH  at 80 °C | 0.5/1 | 1.74/1.86 | 0.5 | 300 | *Adv. Mater. 2024, 36, 2411134* |
| MoNi_4_/MoO_2_ \|\| Ni_2_P @ FePO_x_H_y_ | 1.0 M KOH  at 60 °C | 1 | 1.84 | 0.5 | 72 | *Appl. Catal. B*  *2022, 306, 121127* |
| NiFeCo \|\| NiFe_2_O_4_ | 1.0 M KOH  at 80 °C | 0.88 | 2.2 | 0.1 | 550 min | *J. Mater. Chem. A, 2022, 10, 8401-8412* |
| NiMPL-PTL \|\| NiMPL-PTL | Pure water  at 60°C | 0.5 | 1.90 | - | - | *Joule 2021, 5, 1776-1799* |
| Co_3_S_4_ \|\| NiCoFe-B_i_ | 1.0 M KOH  at 60 °C | 0.5 | 1.98 | 0.5 | 101 | *Int. J. Hydrogen Energy 2024, 59, 1297-1304* |
| Ni-Fe-Co \|\| Ni-Fe-Co | 1.0 M KOH  at 60 °C | 1 | 1.90 | 1 | 100 | *Rsc Adv*  *2020,10, 37429-37438* |
| eCCO/X37-50 \|\| eCCO/X37-50 | 1.0 M KOH  at 45 °C | 1.07 | 1.80 | 0.5 | 500 | *ACS Energy Lett. 2022 7, 8, 2576-2583* |
| NiFe_2_O_4_/NFP-CCS \|\| CuNiFe-LDH/NFP | 1.0 M KOH  at 70 °C | 0.974 | 1.85 | 0.6 | 120 | *Appl. Catal. B 2024, 340, 123187* |
| NiCoP_v_ @NF \|\| NiCoP_v_ @NF | 1.0 M KOH  +seawater | 0.5 | 1.93 | 0.5 | ˃110 | *Adv. Energy Mater. 2024, 2400975* |
| B,V-Ni_2_P \|\| NiFeOOH | 1.0 M KOH  at 60 °C | 1 | 1.92 | 0.5 | 100 | *Small*  *2023, 19, 2208076* |
| Pt/C \|\| CoO_x_-Hf2.5 | 1.0 M KOH  at 75 °C | 1 | 2.28 | 2 | 40 | *ACS Energy Lett. 2024, 9, 1025-1034* |
| NiCoO-NiCo/C \|\| CuCoO | 1.0 M KOH  at 50 °C | 0.504 | 1.85 | 0.44 | 150 | *Appl. Catal. B 2021, 292, 120170* |
| Pt/C \|\| NiCoFe-NDA | 1.0 M KOH  at 50 °C | 0.325 | 1.80 | 0.4* | ~75* | *Energy Environ.*  *Sci. 2021, 14, 6546* |
| Co_2_P-Ni_12_P_5_ \|\| Fe_2_P-Ni_12_P_5_ | 1.0 M KOH  at 60 °C | 1 | 1.95 | - | - | *ACS*  *Sustain. Chem. Eng. 2022, 10, 30, 9956-9968* |
| CoN/VN@NF \|\| P-CoVO@NF | 1.0 M KOH  at 70 °C | 0.5/1 | 1.76/1.84 | 0.5 | 1000 | *Adv. Mater.*  *2024, 36, 2408634* |
| NiPt@NVOC \|\| RuO_2_ | 1.0 M KOH  at 60 °C | 1 | 1.84 | 0.5 | 1000 | *Appl. Catal. B 2025, 363, 124801* |
| Mo_5_N_6_/Ni-450 \|\| Mo_5_N_6_/Co-450 | 1.0 M KOH  at 70 °C | 0.45 | 1.8 | 0.45 | 120 | *Adv. Funct. Mater. 2024, 2412979* |
| Pt/C \|\| Co_3_O_4_ | 1.0 M KOH  at 57 °C | 0.3 | 1.85 | 0.5 | 20 | *Adv. Mater.*  *2022, 34, 2203033* |
| 3-Co_3_S_4_ NS/NF \|\| Cu_0·81_Co_2·19_O_4_ NS/NF | 1.0 M KOH  at 50 °C | 0.431 | 2.0 | 0.5 | ~700* | *Int. J. Hydrogen*  *Energy 2020, 45, 36-45* |
| **Pt-CoP@CNTs/CeO_2_-CoP@NCs \|\| CoP@CNTs/CeO_2_-CoP@NCs** | **1.0 M KOH**  **at 60 °C** | **0.5/1/2** | **1.71/1.84/2.04** | **1** | **1000** | ***This work*** |

* The value is estimated from the original curves shown in the literature.

Reference:

[1] A. S. Botana, M. R. Norman, Phys. Rev. Mater., 2019, 3, 044001.

[2] G. Kresse, J. Hafner, J. of Phys. Condens. Matt. 1994, 6, 8245.

[3] P. E. Blöchl, O. Jepsen, O. K. Andersen, Phys. Rev. B 1994, 49, 16223.

[4] S. Ehrlich, J. Moellmann, W. Reckien, T. Bredow, S. Grimme, ChemPhysChem 2011, 12, 3414.

[5] S. Aleem, V. H. Hoa, and D. H. Kim, J. of Energy Chem. 103 (2025) 264–273.
